# Single-Cell Sequencing Reveals the Heterogeneity of Glioma and Identifies IGFBP2 as A Potential Therapeutic Target

**DOI:** 10.32604/or.2026.079221

**Published:** 2026-06-16

**Authors:** Jian-Lei Kang, Yu-Jie Xu, Qi-Tai Zhao, Bing Zhang, Xin Xu, Bo Yang

**Affiliations:** 1 Department of Neurosurgery, the First Affiliated Hospital of Zhengzhou University, Zhengzhou, China; 2 Department of Neurosurgery, the Affiliated Cancer Hospital of Zhengzhou University, Zhengzhou, China; 3 Department of Oncology, Henan Provincial People’s Hospital, Zhengzhou, China; 4 Biotherapy Center and Cancer Center, the First Affiliated Hospital of Zhengzhou University, Zhengzhou, China; 5 Department of Pathology, the Affiliated Cancer Hospital of Zhengzhou University, Zhengzhou, China

**Keywords:** Glioma, heterogeneity, immunosuppressive tumor microenvironment, insulin-like growth factor binding protein 2

## Abstract

**Background:** Glioma is among the most malignant brain tumors, and its heterogeneity contributes significantly to treatment failure. Comprehensive profiling of cellular and molecular heterogeneity across different glioma stages and recurrence states is crucial for understanding therapeutic resistance and identifying novel targets. Accordingly, this study sought to systematically characterize the cellular and molecular heterogeneity of glioma across different stages and recurrence states using single-cell RNA sequencing, and to identify prognostic subtypes and potential therapeutic targets. **Methods:** We integrated public single-cell RNA sequencing data from glioma specimens, including lower-grade glioma (LGG), glioblastoma (GBM), and paired primary and recurrent tumors. Using these datasets, we identified distinct cellular subpopulations and their molecular signatures. Based on these glioma cell subpopulations, we reclassified gliomas from The Cancer Genome Atlas (TCGA) database into molecular subtypes and constructed a prognostic model. The functional role of a key candidate gene, insulin-like growth factor binding protein 2 (IGFBP2), was validated using *in vitro* knockdown experiments in mouse and human tumor cells and *in vivo* therapeutic studies in murine models, including combination therapy with anti-programmed cell death protein 1 (anti-PD-1) immune checkpoint blockade. **Results:** This analysis revealed that T cells in GBM and recurrent samples were predominantly exhausted, characterized by upregulation of PD-1 and T-cell immunoglobulin and mucin-domain containing^−3^ (Tim3), while myeloid cells exhibited an immunosuppressive phenotype with elevated expression of macrophage migration inhibitory factor (MIF) and cluster of differentiation (CD)276. We identified 12 distinct glioma cell subpopulations with varying proliferative and hypoxic signatures. Based on these subpopulations, TCGA gliomas were reclassified into two major subtypes. One subtype, enriched with myeloid-derived suppressor cells (MDSCs) and regulatory T cells (Tregs), was associated with poorer patient prognosis. A prognostic model was successfully established using differentially expressed genes between the subtypes. Furthermore, IGFBP2 was highly expressed in glioma cells, and its expression negatively correlated with T cell infiltration. *In vitro* knockdown of IGFBP2 downregulated programmed death-ligand 1 (PD-L1) expression on tumor cells. *In vivo*, IGFBP2 knockdown significantly suppressed tumor growth (*p* < 0.01) and extended survival in tumor-bearing mice (*p* < 0.05), and its combination with anti-PD-1 therapy markedly enhanced antitumor efficacy. **Conclusions:** This study provides deeper insights into the cellular ecosystem and heterogeneity of glioma, linking specific cellular features to patient prognosis. We identify IGFBP2 may play a role in regulating immunosuppressive tumor microenvironment and a potential therapeutic target.

## 1 Introduction

Gliomas represent the predominant form of primary neoplasms affecting the central nervous system. These malignancies originate from glial cell lineages or their progenitor cells, subsequently classified into various subtypes [1,2,3]. Due to their critical location within the central nervous system, gliomas are associated with extremely high mortality and poor clinical outcomes [4,5]. According to clinical studies, the median survival time for low-grade gliomas can reach 11.7 years. However, for high-grade gliomas, especially Grade 4 tumors, the median survival time is only about 15 months [6,7].

The treatment strategy for adult gliomas is fundamentally linked to their diagnostic classification [8,9,10]. According to the revised 4th edition of the WHO 2016 classification system, adult diffuse gliomas are initially grouped by microscopic appearance while incorporating key genetic indicators. Four major categories are recognized: diffuse astrocytic tumors, characterized by isocitrate dehydrogenase (IDH) mutation and absence of 1p/19q co-deletion (WHO Grade II–IV); oligodendroglial tumors, defined by IDH mutation and 1p/19q co-deletion (WHO Grade II–IV); glioblastoma (GBM), IDH-wildtype (WHO Grade IV); and diffuse midline glioma, H3K27M-mutant (WHO Grade IV). The tumors classified as Grade II are referred to as low-grade gliomas (LGG) [11]. The 2021 WHO classification establishes a new overarching category of “adult-type diffuse gliomas” for tumors that typically occur in adults and are driven by specific molecular alterations. The current WHO classification of adult-type diffuse gliomas comprises three principal molecularly defined entities: Astrocytoma with IDH mutation (classified across CNS WHO grades II to IV); oligodendroglioma featuring both IDH mutation and 1p/19q codeletion (grades II through III); along with glioblastoma (GBM) that lacks IDH mutation (consistently categorized as grade IV) [12].

Traditional classification strategies have largely relied on bulk tumor sequencing and clinical parameters, which obscures intratumoral heterogeneity [13,14,15]. However, recent advances in single-cell technologies now allow dissection of cellular states at a single-cell level, particularly well-demonstrated in GBM. This approach provides deeper insights into glioma taxonomy and helps identify potential therapeutic targets [16,17,18]. Traditionally, GBM has been classified into four transcriptional subtypes: classical, mesenchymal, proneural, and neural. These subtypes exhibit distinct survival outcomes and differential sensitivities to therapeutics [19,20]. For instance, the mesenchymal subtype is more frequently observed in recurrent GBM, whereas the primary tumor of the same patient may present as proneural or classical. Such subtype heterogeneity often correlates closely with clinical prognosis [21]. However, single-cell RNA sequencing analyses have revealed that cellular populations corresponding to all these subtypes coexist within individual GBM tumors, and tumor cells exhibit significant heterogeneity in gene expression profiles related to the cell cycle and hypoxia response [22,23]. Recent research utilizing single-cell analysis techniques has identified GBM as comprising four unique cellular phenotypes: oligodendrocyte progenitor cell-resembling (OPC-similar), neural progenitor cell-mimicking (NPC-analogous), astrocytic-patterned (AC-comparable), and mesenchymal-featured (MES-characteristic) [24].

The standard of care for gliomas consists of maximal safe surgical resection followed by radiotherapy plus temozolomide (TMZ). This dual-modality approach typically commences within one month following the surgical procedure [25,26]. In addition, bevacizumab—a recombinant humanized monoclonal antibody targeting vascular endothelial growth factor (VEGF)—is approved for the treatment of GBM [27,28]. Tumor-Targeting Fields (TTF), a specialized electromagnetic treatment modality, selectively interferes with rapidly dividing cells by applying low-voltage, mid-frequency alternating currents, serving as a crucial component in GBM therapeutic protocols [29,30]. Recent clinical research has increasingly focused on immunotherapeutic approaches, especially those involving immune checkpoint blockade mechanisms directed against specific molecular targets such as PD-1 (programmed cell death protein 1), PD-L1 (programmed death-ligand 1), and CTLA-4 (cytotoxic T-lymphocyte-associated protein 4) [31]. These therapeutic strategies have undergone rigorous assessment in numerous experimental studies concerning glioma treatment [32,33]. A multicenter randomized study examined immunological reactions and survival rates in GBM patients receiving preoperative pembrolizumab therapy. Findings demonstrated that administering pembrolizumab before surgery amplified tumor-specific immune activation at both regional and systemic levels [34]. Additionally, a phase III clinical investigation (NCT02017717) evaluated the therapeutic potential and safety profile of combining nivolumab with ipilimumab for recurrent GBM. Results demonstrated that this dual immunotherapy approach failed to enhance overall survival rates among enrolled participants [35]. Immune checkpoint therapies primarily function by activating the host immune system, which necessitates a deeper understanding of the glioma tumor microenvironment. At the single-cell level, recent studies have revealed that GBM exhibits one of the most immunosuppressive tumor microenvironments, characterized by abundant infiltration of tumor associated macrophages and regulatory T cells, a critical factor underlying the limited success of glioma immunotherapy in clinical trials [36,37]. Single-cell analysis revealed the heterogeneity of immune cells across different spatial regions in GBM [38].

Therefore, in this study, we aimed to comprehensively delineate the cellular heterogeneity of glioma at single-cell resolution, further refine molecular subtypes based on tumor cell subpopulations, and explore key regulators of the immunosuppressive tumor microenvironment that may serve as potential therapeutic targets.

## 2 Materials and Methods

### 2.1 Ethics Statement

The study was performed with the approval of the Ethics Committee of Henan Cancer Hospital (No. 2022-312). Clinical samples were collected from patients after written informed consent was obtained. All animal experiments were performed in accordance with the guidelines approved by the Animal Care and Use Committee of the Experimental Animal Center of Zhengzhou University (ZZU-LAC20220225[09]). All authors reviewed and approved the final manuscript.

### 2.2 Single-Cell Data Processing and Cell Classification

The datasets GSE138794 and GSE182109 were initially acquired from the GEO repository (https://www.ncbi.nlm.nih.gov/geo/) [39,40]. Each dataset underwent independent processing through the Seurat package (version 5.3.0). A Seurat object was created using the *CreateSeuratObject* function with the parameters min.cells = 3 and min.features = 200. The two datasets were subsequently merged using the merge function in Seurat. Subsequently, cells were further filtered based on the following criteria to retain high-quality cells: percentage of mitochondrial genes < 20%, nCount_RNA between 500 and 150,000, nFeatures_RNA between 400 and 10,000, and log10 GenesPerUMI > 0.8. The dataset underwent normalization through the NormalizeData procedure with preset configurations. Highly variable features were identified by selecting the top 3000 most fluctuating genes, followed by data scaling via the ScaleData operation. Principal component analysis (PCA) was employed for dimensionality reduction, utilizing 50 principal components (PCs) for subsequent clustering. The harmony package (v1.2.0) was implemented with the RunHarmony function to mitigate batch-related variations. Cellular relationships were mapped using FindNeighbors (dimensional range 1–20), while two-dimensional visualization was achieved through Uniform Manifold Approximation and Projection (UMAP) embedding. Marker genes for each cluster were identified with the FindAllMarkers function in Seurat (method = “Wilcoxon”), which detects genes significantly upregulated in one cluster compared to all others, serving as potential cell-type markers. Cell types were manually annotated by cross-referencing the identified marker genes with known canonical cell-type markers.

### 2.3 Calculation of Pathway Enrichment Scores across Cellular Subsets

To evaluate pathway across distinct cellular subgroups, we initially obtained the Hallmark gene collections from the MSigDB database (https://www.gsea-msigdb.org/gsea/msigdb). Cellular subgroup-specific gene evaluations set were performed employing Seurat’s AddModuleScore functionality. Visualization of pathway activation trends among various cell populations was achieved through heatmap construction using the pheatmap (version: 1.0.12) toolkit in R. The resulting heatmap displays pathways along the vertical axis and cellular classifications along the horizontal dimension, where color saturation corresponds to standardized pathway activation values (Z-score transformed).

### 2.4 Detection of Differentially Expressed Genes and Functional Pathway Analysis

To pinpoint genes exhibiting differential expression (DEGs) across the two cellular populations, we employed the Seurat package’s (version 5.3.0) FindMarkers function, utilizing the Wilcoxon rank-sum test for statistical evaluation. Genes were designated as differentially expressed based on stringent criteria: a log2 fold change exceeding 0.5, an adjusted *p*-value (*p*_val_adj) below the 0.05 and expressing percentage more than 15%. Genes demonstrating avg_log2FC values greater than 0.5 were categorized as upregulated, while those with values below −0.5 were classified as downregulated. The resulting DEGs were graphically represented through a volcano plot generated with the ggplot2 package (version 3.4.4). Following this, we performed functional enrichment analyses on the upregulated and downregulated gene sets independently. Using the clusterProfiler package, we examined Gene Ontology (GO) terms and Kyoto Encyclopedia of Genes and Genomes (KEGG) pathways. Only terms and pathways meeting dual significance criteria—both *p*-value and q-value below 0.05—were deemed substantially enriched.

### 2.5 Survival Analysis Based on Cell Subpopulations

We obtained the RNA-seq data (in TPM format) and corresponding clinical information for GBM and LGG from The Cancer Genome Atlas (TCGA) database (https://portal.gdc.cancer.gov/). Additionally, glioma RNA-seq data (in FPKM format) and matched clinical data were acquired from the Chinese Glioma Genome Atlas (CGGA) database (https://www.cgga.org.cn/download.jsp). To define cell type-specific gene signatures, we performed differential expression analysis using the FindAllMarkers function (Wilcoxon rank-sum test) in the Seurat package (version 5.3.0). For each cell type, genes that were significantly upregulated compared to all other cell populations (with a log2 fold-change > 1) were selected to represent its characteristic gene set. Subsequently, the single-sample Gene Set Enrichment Analysis (ssGSEA) algorithm was employed to calculate an enrichment score for each cell type signature in every bulk tumor sample, representing the relative abundance or activity of that specific cell population. To evaluate the prognostic relevance of each identified cell type, Kaplan-Meier survival analysis was conducted. For the ssGSEA score of each cell type, patients within a cohort were dichotomized into high- and low-score groups using the median score as the cutoff. Survival curves were plotted for the two groups, and the statistical significance of the difference in survival distribution was assessed using the two-sided log-rank test, with a *p*-value < 0.05 considered significant.

### 2.6 Immune Estimation of Two Subtypes of Glioma

To comprehensively characterize immune cell infiltration within the tumor microenvironment, we used two distinct computational approaches for immune infiltration profiling: MCP-counter alongside single-sample Gene Set Enrichment Analysis (ssGSEA). Through the MCP-counter technique, we measured precise abundance metrics for eight distinct immune cell types in addition to two stromal cell categories. This approach relies on the expression of cell-type-specific marker genes, and its output exhibits a linear relationship with actual cell counts, making it suitable for cross-sample comparisons. Simultaneously, we applied the ssGSEA algorithm to compute an alternative immune infiltration score based on human immune-state signature gene sets. ssGSEA generates an enrichment score associated with the activity of specific immune cell types or functional states by rank-ordering the member genes of predefined immune gene sets within each sample. All analyses were performed in the R environment. The MCP-counter analysis was implemented using its official R package (version 1.2.0), and ssGSEA was carried out via the GSVA R package (version 1.44.0).

### 2.7 Consensus Clustering of Glioma Based on Tumor Cell Subpopulations

We first integrated the cell-type signature enrichment score matrices for GBM and LGG from The Cancer Genome Atlas (TCGA). To stratify gliomas based on tumor cell subpopulations, genes with a log2 fold-change greater than 1 for each tumor cell subpopulation were selected to represent its respective marker set. Concurrently, batch effects between LGG and GBM mRNA expression data from TCGA were corrected using the empirical Bayes-based ComBat algorithm. Subsequently, the single-sample Gene Set Enrichment Analysis (ssGSEA) method was applied to compute enrichment scores for each cell subpopulation in every sample. Using the integrated dataset of GBM and LGG tumor samples, unsupervised molecular subtyping was performed with the ConsensusClusterPlus R package (version 1.72.0). This method employs repeated resampling and clustering to generate a consensus matrix. To evaluate the prognostic significance of the identified molecular subtypes, Kaplan–Meier survival curves were plotted. Survival probabilities were calculated using the *survfit* function from the survival package, and differences in overall survival between subtypes were compared via the log-rank test implemented by the *survdiff* function. A significance level of *p* < 0.05 was established for statistical evaluation. To investigate the molecular distinctions between subtypes more thoroughly, comparative expression profiling was performed utilizing the limma package (version 3.52.1) in R. Transcriptional data underwent linear modeling through the *lmFit* functionality, succeeded by empirical Bayesian adjustment of error terms using the *eBayes* method to enhance variance reliability. Genes exhibiting differential expression were filtered based on these criteria: absolute logarithmic fold-change (|logFC|) exceeding 1 and Benjamini-Hochberg corrected significance (adj.*p*.Val) below 0.05.

### 2.8 Construction and Validation of the Prognostic Model

To construct prediction model, the CGGA database served as the primary training cohort. Initial screening involved conducting individual Cox regression assessments on genes exhibiting differential expression patterns across distinct glioma classifications, identifying those with statistically meaningful survival correlations (significance threshold *p* < 0.05). We then implemented LASSO-based variable selection methodology to refine the gene pool and isolate the optimal predictive gene cluster. The final stage involved establishing a multi-factor Cox proportional risk model incorporating these selected genetic markers. To verify the stability and broader applicability of prognostic indicator, we performed cross-validation using the combined TCGA repository (encompassing both GBM and LGG cases) along with an external validation cohort (GSE43378) sourced from GEO. Additionally, we generated ROC curve analyses to quantify the model’s predictive performance across 1-year, 3-year, and 5-year intervals.

### 2.9 Construction of Stable IGFBP2 Knockdown and Overexpression Cell Lines

The shRNA sequences targeting human IGFBP2 and mouse Igfbp2, along with the corresponding lentiviral supernatants, were synthesized by Shanghai Sangon Biotech. The target sequence for human IGFBP2 was 5′-CCGGGCCTTCATCAAGAACATCAA-3′, and that for mouse Igfbp2 was 5′-GCCTGTAAGACCGTCTACTAT-3′. A non-targeting scramble shRNA lentivirus was used as the negative control, and untransduced cells were used as blank controls during puromycin selection. For overexpression, lentiviral vectors encoding full-length human IGFBP2 or mouse Igfbp2 cDNA, along with corresponding empty vector controls, were also constructed and packaged by Shanghai Sangon Biotech. Prior to the main experiments, the puromycin sensitivity of both U87 and GL261 cell lines was determined. The human glioma cell line U87 and the mouse glioma cell line GL261 were obtained from Wanwushengwu Biotechnology (Hefei, China; Cat#Delf-10592 for U87 and Cat#Delf-15805 for GL261). All cell lines were routinely tested and confirmed to be free of mycoplasma contamination using a mycoplasma detection kit. In addition, STR profiling was performed to authenticate the identity of the U87 and GL261 cell line. Cells were seeded in 24-well plates, and after 24 h, the culture medium was replaced with fresh complete medium containing a puromycin concentration gradient (0.5–3.0 μg/mL). Cell viability was monitored daily, and the minimal concentration that achieved complete cell death within 3–5 days was defined as the optimal working concentration (2.5 μg/mL for U87 and 1.5 μg/mL for GL261 cells). For lentiviral transduction, U87 and GL261 cells were seeded at a density of 2 × 10^5^ cells per well in 6-well plates one day prior to infection, reaching 30–50% confluency. Lentiviral stocks (including IGFBP2/Igfbp2 knockdown, overexpression constructs, and their respective controls) were thawed on ice, while complete medium was pre-warmed to 37°C. Cells were washed once with PBS, and viral particles were added in antibiotic-free medium supplemented with polybrene (5 μg/mL). Lentiviral transduction was performed at an optimized MOI determined in preliminary experiments to achieve high transduction efficiency with minimal cytotoxicity. The same viral input and transduction conditions were applied across knockdown, overexpression, and corresponding control groups to ensure consistency. After 8–12 h, the medium was replaced with fresh complete medium. Puromycin selection was initiated 72 h post-infection and maintained for 5–7 days, with medium refreshed every two days, until all cells in the untransduced control wells were eliminated and resistant colonies were clearly observed. The efficiency of IGFBP2 knockdown and overexpression was validated after selection by measuring protein expression levels using Western blotting. Only cells with stable and significant downregulation or upregulation of IGFBP2 compared with respective controls were used for subsequent experiments.

### 2.10 Western Blot Analysis of IGFBP2 and STAT3 Expression

Following enzymatic dissociation with trypsin, cellular material was harvested via centrifugation and subsequently disrupted in protein extraction buffer supplemented with protease inhibitors to isolate total protein content. Protein quantification was performed employing the bicinchoninic acid (BCA) assay methodology. For electrophoretic separation, aliquots containing 25 μg of protein per specimen were subjected to sodium dodecyl sulfate-polyacrylamide gel electrophoresis (SDS-PAGE) analysis (utilizing a 10% resolving gel matrix). Post-electrophoresis, protein bands were electrotransferred onto polyvinylidene difluoride (PVDF) membranes through conventional wet transfer protocols. Membrane blocking was conducted with 5% bovine serum albumin (BSA) solution under ambient conditions for 60 min, succeeded by overnight exposure at 4°C to primary antibodies targeting IGFBP2 (dilution 1:1000, Thermo Fisher Scientific, Waltham, MA, USA, Cat#PA5-79450), STAT3 (dilution 1:1000, Abcam, Cambridge, UK, Cat#ab119352), p-STAT3 (dilution 1:1000, Abcam, Cambridge, UK, Cat#ab267373) and β-actin (dilution 1:5000, Thermo Fisher Scientific, Waltham, MA, USA, Cat#MA5-15739). Following temperature equilibration for one hour at room temperature, membranes were probed with appropriate secondary antibodies for an equivalent duration. After triple-rinsing with Tris-buffered saline containing Tween-20 (TBST), chemiluminescent detection was performed using ECL reagent, with signal acquisition accomplished via the GelView 5000 Plus imaging platform (manufactured by Guangzhou Biolight Biotechnology Co., Ltd.).

### 2.11 Flow Cytometric Analysis of PD-L1 Expression

U87 and GL261 cultures were harvested during exponential growth phase following enzymatic dissociation with trypsin-EDTA solution, with subsequent inactivation using complete growth medium. Following centrifugal separation, cellular suspensions underwent two washing cycles with chilled phosphate-buffered saline (PBS). To minimize nonspecific antibody interactions, cell aggregates were pre-treated with species-specific CD16/32 blocking antibodies (human-specific for U87, murine-specific for GL261) at 4°C for a quarter-hour. Cell aliquots (1 × 10^6^ cells) were prepared in 100 μL antibody dilution buffer (PBS supplemented with 2% FBS) for immunolabeling procedures. U87 specimens received PE-labeled anti-human CD274 (B7-H1) monoclonal antibody (29E.2A3 clone, BioLegend, San Diego, CA, USA, Cat#329705), whereas GL261 samples were treated with PE-conjugated anti-mouse CD274 (10F.9G2 clone, BioLegend, San Diego, CA, USA Cat#124307). Antibody incubation proceeded for 30 min at refrigerated temperatures under light-protected conditions, followed by dual buffer washes to eliminate unbound reagents. Processed cells were finally reconstituted in 300 μL PBS for immediate analytical processing. Flow cytometric evaluation utilized a DxFLEX platform (Beckman Coulter, Brea, CA, USA) with CytExpert software for data capture. Viable cellular subpopulations were identified through forward/side scatter profiling. PD-L1 expression thresholds were established relative to isotype controls, with subsequent quantification of both positive cell percentages and median fluorescence intensity values.

### 2.12 Multiplex Immunofluorescence Staining

The simultaneous detection of IGFBP2 and CD8^+^ T cells was achieved through multiplex immunofluorescence staining employing Tyramide Signal Amplification (TSA) methodology. Tissue specimens embedded in paraffin underwent sequential processing including deparaffinization, rehydration, and antigen unmasking procedures. The initial antibody incubation sequence involved two distinct phases. Tissue sections first underwent prolonged exposure to a rabbit-derived IGFBP2-targeting primary antibody (Thermo Fisher Scientific; Waltham, MA, USA, Cat#PA5-79450, diluted 1:200) at 4°C for approximately 16 h. Subsequently, specimens were treated for half an hour at ambient temperature using a polymer-based secondary antibody conjugated with horseradish peroxidase, specifically designed for rabbit immunoglobulin detection. IGFBP2 protein was visualized using TYR-570 fluorescent dye (emission wavelength 570 nm, red fluorescence) via TSA development. Subsequently, microwave-mediated heat-induced epitope retrieval was performed to strip the antibody complexes from the first round. For the second cycle, slides were incubated with rabbit anti-human CD8α primary antibody (1:100, Abcam, Cambridge, UK, Cat#ab237709), followed by the same horseradish peroxidase (HRP)-conjugated secondary antibody. CD8^+^ T cells were labeled using TYR-620 fluorescent dye (emission wavelength 620 nm, orange-red fluorescence) via TSA development. Finally, nuclei were counterstained with DAPI, and sections were mounted with an anti-fade mounting medium. Stained slides were imaged and analyzed using a fluorescence or confocal microscope.

### 2.13 In Vivo Mouse Experiments

All animal experiments were approved by the Institutional Animal Care and Use Committee (IACUC) of Zhengzhou University (ZZU-LAC20220225[09]) and were conducted in accordance with relevant guidelines and regulations. The mouse glioma cell line GL261 cells in the logarithmic growth phase were trypsinized, resuspended and washed twice with pre-cooled sterile PBS, and finally adjusted to a density of 5 × 10^6^ cells/mL in PBS for subsequent use. Female C57BL/6J (4–6 weeks old, 12–25 g) mice were purchased from SPF Biotechnology Co., Ltd. (Beijing, China). Once tumors became palpable (approximately 7 days post-inoculation), mice were stratified by tumor volume and randomly assigned to four groups: (1) control group (n = 5), (2) knockdown group (n = 5), (3) control + anti-PD-1 group (n = 5), and (4) knockdown + anti-PD-1 group (n = 5). For inoculation, each mouse was subcutaneously injected in the right flank with 100 μL of cell suspension (5 × 10^5^ cells per mouse) using a 1 mL insulin syringe. Regarding treatment, the two anti-PD-1 treatment groups received intraperitoneal injections of 200 μg anti-PD-1 monoclonal antibody (clone RMP1-14, Bio X Cell, Lebanon, NH, USA; Cat# BE0146) on days 7, 10, and 13 post-inoculation, while the control groups were injected with an equal volume of PBS. Starting from day 7 post-inoculation, tumor length (L) and width (W) were measured every 3 days using a digital caliper, and tumor volume was calculated using the formula V = (L × W^2^)/2. Body weight was monitored consistently throughout the experiment. Euthanasia was performed when any of the following endpoints was reached: tumor volume exceeding 2000 mm^3^, ulceration or infection of the tumor, body weight loss exceeding 20% of the initial weight, or severe impairment of activity. Survival time was recorded from the day of inoculation to the day of euthanasia.

### 2.14 Statistical Analysis

Data processing was performed using with R (version 4.0.1, R Foundation for Statistical Computing, Vienna, Austria) for computational biology datasets and GraphPad Prism (version 8.3.0, GraphPad Software, San Diego, California, USA) for laboratory investigations. Bioinformatics comparisons utilized the nonparametric Wilcoxon rank-sum approach (Mann-Whitney U test), while experimental group contrasts employed independent two-sample *t*-tests. Survival outcomes were assessed through Kaplan-Meier estimation, with group disparities examined via log-rank testing. Statistical significance was set at *p* < 0.05 for all analyses.

## 3 Results

### 3.1 The Cellular Composition and Heterogeneity of Glioma

**Figure 1:**
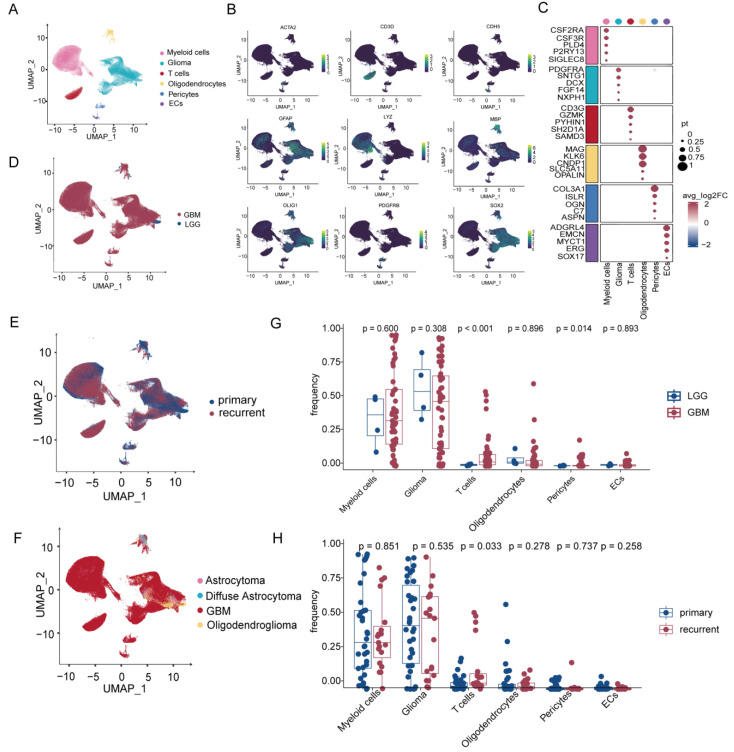
Single-cell atlas of the glioma microenvironment. (**A**) Uniform Manifold Approximation and Projection (UMAP) visualization of major cell types from glioma samples. (**B**) UMAP showing expression of canonical marker genes across major cell types. (**C**) Dot plot showing specific marker genes of major cell types. (**D**–**F**) UMAP revealing the major cellular composition of (**D**) glioblastoma multiforme (GBM) and low-grade glioma (LGG); (**E**) primary and recurrent tumor tissues; (**F**) different tissue types. (**G**,**H**) Box plots showing differences in subset proportions between (**G**) LGG and GBM; and between (**H**) primary and recurrent tumors.

To delineate the heterogeneity of glioma and the composition of the tumor microenvironment, we integrated two public single-cell RNA sequencing datasets, which include both LGG and GBM. Following quality control and filtering (Supplementary Fig. S1A–C), a total of 246, 127 cells were retained for downstream analysis. Using unsupervised clustering and UMAP dimensionality reduction, we identified six major cell types. Based on known marker genes and cluster-specific signature genes, these subpopulations were annotated as myeloid cells, glioma cells, T cells, pericytes, endothelial cells, and oligodendrocytes (Fig. 1A–C). The majority of these cells were derived from GBM tissues (Fig. 1D), with 225,392 cells originating from GBM and 20,735 from LGG. Among them, 151,707 cells were from primary tumors, while 94,420 cells were from recurrent tumors (Fig. 1E). Histopathological analysis also revealed that these cells were predominantly derived from GBM tumor tissues (Fig. 1F). Subsequent analysis of cellular proportions across different samples revealed that T cells and pericytes were more abundant in GBM-derived tissues (Fig. 1G). Moreover, T cell infiltration was significantly higher in recurrent tumor samples (Fig. 1H). These results reveal the cellular composition of the glioma microenvironment.

### 3.2 T Cells in Gbm Exhibited an Exhausted Phenotype

**Figure 2:**
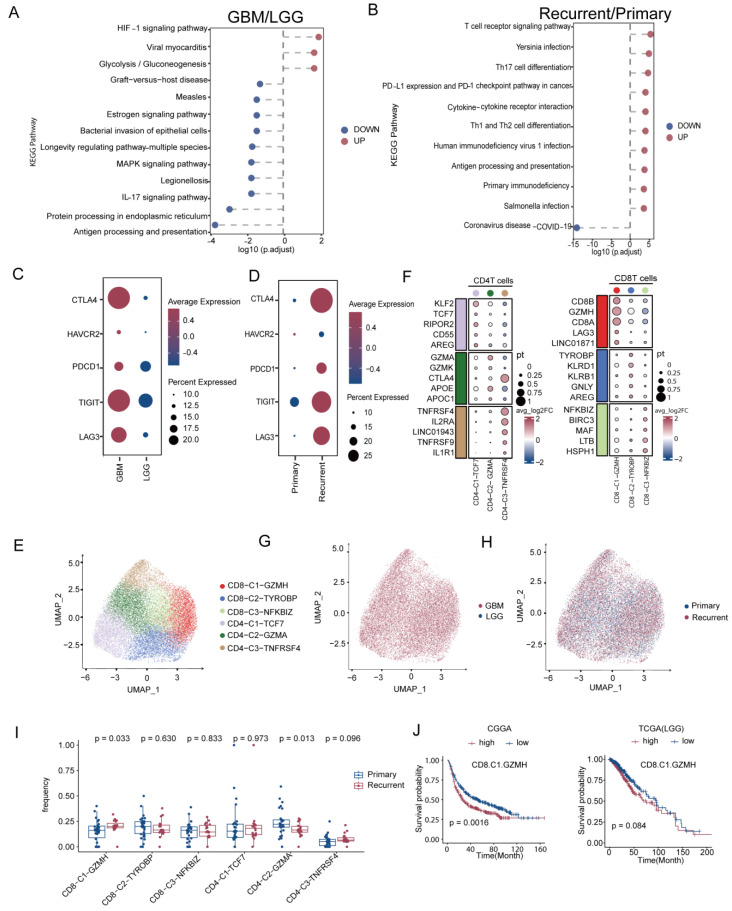
T cells in glioblastoma multiforme (GBM) and recurrent gliomas exhibit an exhausted phenotype with prognostic significance. (**A**,**B**) Kyoto Encyclopedia of Genes and Genomes (KEGG) pathway enrichment analysis of T cells between (**A**) LGG and GBM; (**B**) primary and recurrent tumor. (**C**,**D**) Dot plot showing exhausted genes between (**C**) low-grade glioma (LGG) and GBM; (**D**) primary and recurrent tumor. (**E**) Uniform Manifold Approximation and Projection (UMAP) showing the T cell clusters. (**F**) Dot plot showing specific marker genes of cluster of differentiation (CD) 4 and CD8^+^ T cell clusters. (**G**,**H**) UMAP revealing the major cellular composition of (**G**) GBM and LGG; (**H**) primary and recurrent tumor tissues. (**I**) Box plots showing differences in subset proportions between primary and recurrent tumors. (**J**) Kaplan-Meier survival analysis revealing the prognostic significance of CD8-C1-granzyme H (GZMH) T cell infiltration in glioma patients using The Cancer Genome Atlas (TCGA) and Chinese Glioma Genome Atlas (CGGA) datasets.

To investigate the phenotypic and functional landscape of T cells within the glioma microenvironment, we first analyzed T cell differences between LGG and GBM, as well as between primary and recurrent tumors. Differential gene expression and enrichment analysis revealed that in GBM, T cell adhesion and activation processes were significantly upregulated compared to those in LGG (Supplementary Fig. S2A,B). Moreover, KEGG pathway enrichment indicated that T cells in GBM exhibited activation of the HIF-1 signaling pathway and the glycolysis pathway (Fig. 2A), both of which are characteristic alterations also observed in tumor cells. Compared to T cells from primary tumor tissues, those from recurrent tumors also displayed a markedly activated state (Fig. 2B, Supplementary Fig. S2C,D). T cells in GBM and recurrent tumors exhibited high expression exhaustion-associated molecules such as PDCD1 (encoding PD-1), HAVCR2 (encoding TIM-3), and CTLA4 (Fig. 2C,D). Subsequent subclustering of T cells identified three distinct CD8^+^ T cell subsets and three CD4^+^ T cell subsets, each expressing unique marker genes (Fig. 2E,F). UMAP visualization showed that the majority of T cells were derived from GBM, with each subset being relatively evenly distributed between primary and recurrent samples (Fig. 2G,H). Further analysis of subset proportions revealed a significant increase in the CD8-C1-GZMH subset in recurrent tumor tissues (Fig. 2I). This subset also exhibited high expression of exhaustion-related genes (Supplementary Fig. S2E), suggesting a functionally exhausted state of T cells in the recurrent tumor microenvironment. Survival analysis based on TCGA and CGGA datasets indicated that a higher signature score of this T cell subset was associated with poorer prognosis in glioma patients (Fig. 2J, Supplementary Fig. S2F).

### 3.3 Characteristics of Myeloid Cells in the Glioma Microenvironment

**Figure 3:**
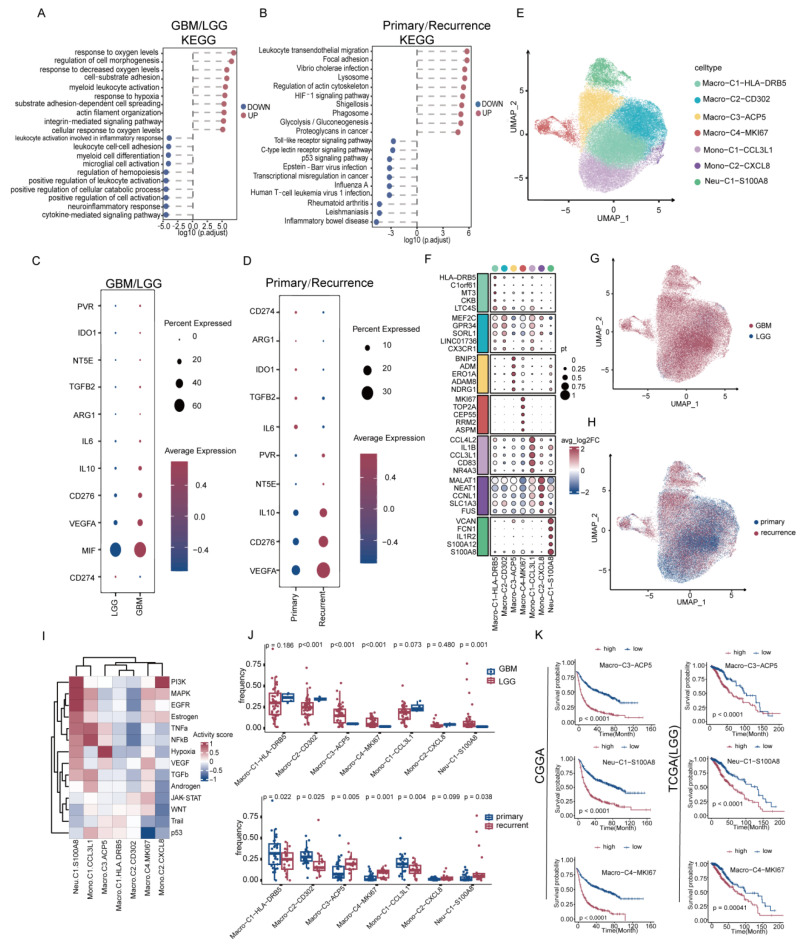
Functional heterogeneity and clinical significance of myeloid cell subsets in the glioma microenvironment. (**A**,**B**) Kyoto Encyclopedia of Genes and Genomes (KEGG) pathway enrichment analysis of myeloid cells between (**A**) low-grade glioma (LGG) and glioblastoma multiforme (GBM); (**B**) primary and recurrent tumor. (**C**,**D**) Dot plot showing suppressive genes between (**C**) LGG and GBM; (**D**) primary and recurrent tumor. (**E**) Uniform Manifold Approximation and Projection (UMAP) showing the myeloid cell clusters. (**F**) Dot plot showing specific marker genes of myeloid cell clusters. (**G**,**H**) UMAP revealing the major cellular composition of (**G**) GBM and LGG; (**H**) primary and recurrent tumor tissues. (**I**) Heatmap showing the signaling activation across myeloid cell subsets. (**J**) Box plots showing differences in subset proportions between (Upper panel) LGG and GBM; (Lower panel) primary and recurrent tumors. (**K**) Kaplan-Meier survival analysis revealing the prognostic significance of infiltration of myeloid cell subsets in glioma patients using The Cancer Genome Atlas (TCGA) and Chinese Glioma Genome Atlas (CGGA) datasets.

Previous studies have revealed that myeloid cells constitute a substantial proportion of the glioma microenvironment, suggesting their critical role in tumor progression. We first analyzed the functional characteristics of myeloid cells in GBM and LGG. In GBM, myeloid cells were found to be involved in leukocyte migration and activation (Supplementary Fig. S3A,B). Pathway enrichment analysis indicated that these cells primarily activate stress responses to oxygen levels (Fig. 3A). By comparing myeloid cells in recurrent and primary tumor tissues, we observed that in the more aggressive recurrent tumors, myeloid cells exhibited a pronounced response to hypoxia (Fig. 3B, Supplementary Fig. S3C,D). Furthermore, in both GBM and recurrent tumors, myeloid cells exhibited a suppressive phenotype characterized by the expression of inhibitory molecules such as MIF, CD276, VEGFA, IL-6, and IL-10 (Fig. 3C,D). To further delineate the heterogeneity of these myeloid cells, we performed subcluster identification and defined four macrophage subclusters, two monocyte subclusters, and one neutrophil subcluster, each expressing unique marker genes (Fig. 3E,F). The majority of myeloid cells originated from GBM (Fig. 3G), and all subclusters were present in both primary and recurrent tumors (Fig. 3H). Characterization of these subclusters revealed that the Neu-C1-S100A8 neutrophil subcluster activated several oncogenic pathways, including EGFR and PI3K, while the Macro-C3-ACP5 subcluster was predominantly associated with hypoxia-related pathways (Fig. 3I). Notably, the proportions of Macro-C3-ACP5, Macro-C4-MKI67, and Neu-C1-S100A8 were significantly elevated in GBM and recurrent tumors compared to LGG and primary tumor tissues (Fig. 3J). Using the TCGA and CGGA databases, we further validated that increased infiltration scores of these three subclusters were correlated with poorer patient prognosis (Fig. 3K, Supplementary Fig. S3E).

### 3.4 Glioma Heterogeneity Was Associated with Tumor Progression

**Figure 4:**
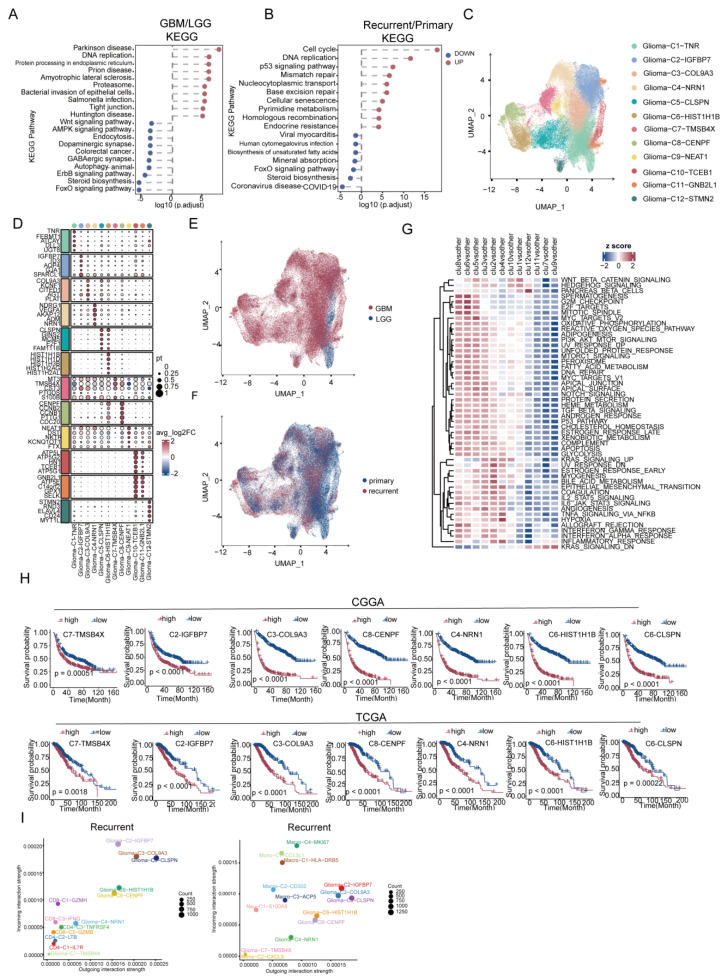
Glioma cellular heterogeneity is associated with tumor progression. (**A**,**B**) Kyoto Encyclopedia of Genes and Genomes (KEGG) pathway enrichment analysis of glioma cells between (**A**) low-grade glioma (LGG) and glioblastoma multiforme (GBM); (**B**) primary and recurrent tumor. (**C**) Uniform Manifold Approximation and Projection (UMAP) showing the glioma cell clusters. (**D**) Dot plot showing specific marker genes of glioma cell clusters. (**E**,**F**) UMAP revealing the major cellular composition of (**E**) GBM and LGG; (**F**) primary and recurrent tumor tissues. (**G**) Heatmap showing the signaling activation across glioma cell clusters. (**H**) Kaplan-Meier survival analysis revealing the prognostic significance of glioma subsets in patients using The Cancer Genome Atlas (TCGA) and Chinese Glioma Genome Atlas (CGGA) datasets. (**I**) Scatter plot analysis revealing communication patterns between glioma subpopulations and T cell/myeloid cell clusters in recurrent tumor.

Tumor cell heterogeneity is a critical factor in tumorigenesis and progression. The analysis revealed that tumor cells derived from GBM and recurrent tumors exhibited activation of pathways related to DNA replication, and cell cycle (Fig. 4A,B, Supplementary Fig. S4A–D). This suggests these cells possess enhanced proliferative capacity, which is consistent with the clinical aggressiveness observed in the corresponding patients. Further subpopulation identification classified these glioma cells into twelve distinct subclusters, each harboring characteristic molecular signatures (Fig. 4C,D). The majority of these tumor cells originated from GBM samples (Fig. 4E). Notably, the C12-STMN2 subpopulation was predominantly derived from primary tumors, implying a unique role for this subtype (Fig. 4F). Pathway analysis indicated that several subpopulations—namely C1-TNR, C2-IGFBP7, C3-COL9A3, C4-NRN1, C5-CLSPN, C6-HIST1H1B, C8-CENPF, and C10-TCEB1—were enriched in multiple oncogenic pathways, including hypoxia, PI3K-AKT, and mTOR signaling. In contrast, the remaining subpopulations showed downregulation of the KRAS signaling pathway (Fig. 4G). We next examined the association between these tumor cell subpopulations and patient survival. This analysis revealed that seven subpopulations (C2–C8) consistently exhibited a significant trend in both the TCGA and CGGA databases: higher infiltration scores were associated with poorer patient prognosis (Fig. 4H, Supplementary Fig. S4E). Furthermore, we investigated the interaction patterns between these subpopulations and T cells as well as myeloid cells. In recurrent tumor tissues, C2-IGFBP7, C3-COL9A3, and C5-CLSPN acted as the primary signal senders, while CD8-C1-GZMH, Macro-C4-MKI67, and Mono-C1-CCL3L1 served as the main receivers (Fig. 4I). In contrast, within primary tumor tissues, C3-COL9A3, C5-CLSPN, and C6-HIST1H1B were identified as the dominant signal senders. Although interactions between these subpopulations and T cells were relatively limited, two myeloid subsets—Macro-C4-MKI67 and Macro−C3−ACP5—emerged as the principal signal recipients (Supplementary Fig. S4F).

### 3.5 Identification of Glioma Subtypes

**Figure 5:**
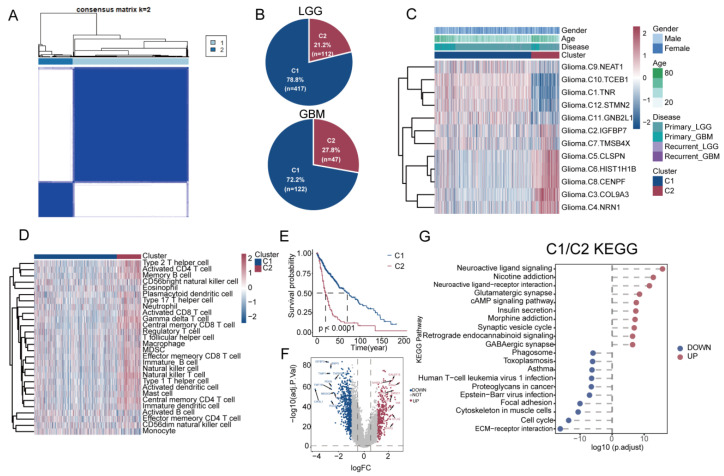
Identification of glioma subtypes. (**A**) Heatmap showing consensus clustering of glioma subtypes in The Cancer Genome Atlas (TCGA). (**B**) Proportional distribution of C1 and C2 subpopulations in low-grade glioma (LGG) and glioblastoma multiforme (GBM). (**C**) Heatmap showing the infiltration of glioma subclusters in C1 and C2 subtypes. (**D**) Heatmap showing the score of immune cell infiltration in C1 and C2 subtypes. (**E**) Kaplan-Meier survival analysis revealing the prognostic significance of C1 and C2 subtypes in TCGA glioma cohort. (**F**) Volcano plot showing the DEGs between C1 and C2 subtypes. (**G**) Kyoto Encyclopedia of Genes and Genomes (KEGG) enrichment analysis of Differentially Expressed Genes (DEGs) between C1 and C2 subtypes.

To address tumor heterogeneity, we redefined glioma subtypes. We first integrated the LGG and GBM datasets from the TCGA database (Supplementary Fig. S5A). Using characteristic genes of each glioma subpopulation identified from single-cell data, we then performed consensus clustering to classify glioma subtypes. This analysis identified two distinct subtypes, designated C1 and C2 (Fig. 5A). Most patients in both LGG and GBM were classified into the C1 subtype (Fig. 5B). To further eliminate the potential confounding effects of intrinsic differences between LGG and GBM on the classification, consensus clustering was performed separately in LGG and GBM cohorts. Consistently, two distinct subtypes were identified in both groups (Supplementary Fig. S5B). The C2 subtype was primarily composed of the C2-IGFBP7, C3-COL9A3, C4-NRN1, C5-CLSPN, C6-HIST1H1B, C7-TMSB4X, and C8-CENPF subpopulations, while C9-NFAT1 was relatively evenly distributed across both subtypes. The remaining subpopulations were predominantly enriched in the C1 subtype (Fig. 5C). Immune profiling revealed that the C2 subtype exhibited an immunologically active phenotype, characterized by higher scores for effector immune cells such as B cells, CD4^+^ T cells, and CD8^+^ T cells, along with elevated levels of immunosuppressive cells including myeloid-derived suppressor cells and regulatory T cells (Fig. 5D, Supplementary Fig. S5C). Moreover, consistent trends between the C1 and C2 subtypes were observed in both the LGG and GBM cohorts when analyzed separately (Supplementary Fig. S5D). Survival analysis showed that patients with the C2 subtype had a poorer prognosis, suggesting that the immune cell state in this subtype may be functionally suppressed (Fig. 5E). Differential expression analysis between the two subtypes revealed that pathways related to chromatin segregation and cell cycle were significantly enriched in the C2 subtype, indicating that tumor cells in this subtype may possess enhanced proliferative capacity (Fig. 5F,G, Supplementary Fig. S5E).

### 3.6 Construction and Validation of the Prognostic Prediction Model

**Figure 6:**
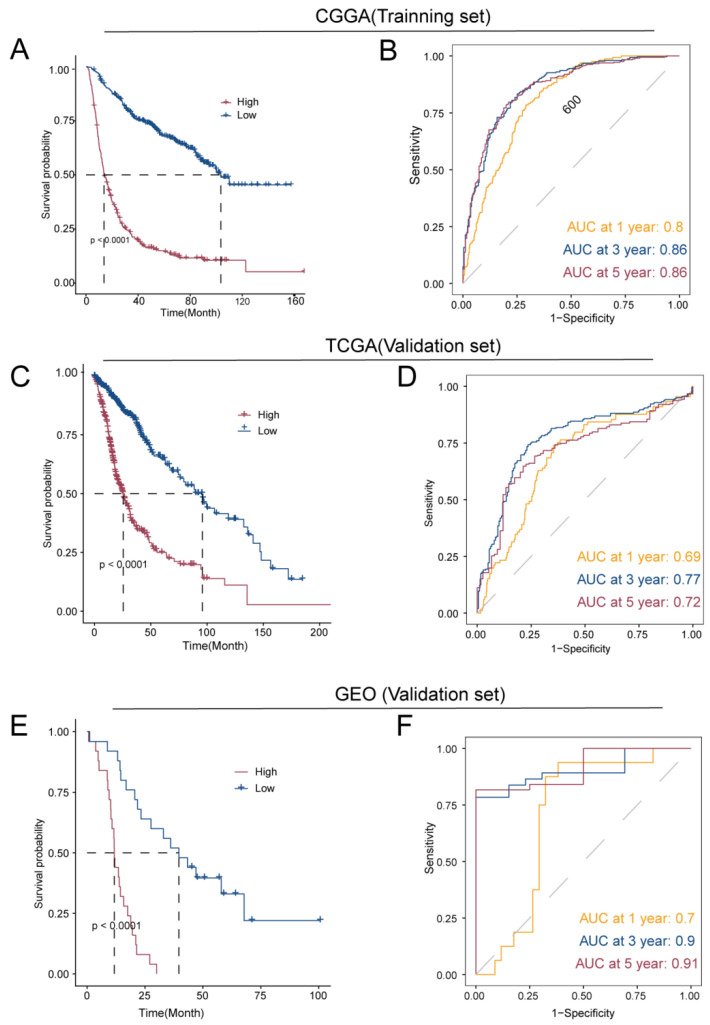
Construction and validation of prognostic model. (**A**) Kaplan-Meier survival analysis revealing the prognostic significance of high risk and low risk group in training set. (**B**) Receiver Operating Characteristic (ROC) analysis of prognostic model at 1, 3, 5 year in training set. (**C**) Kaplan-Meier survival analysis revealing the prognostic significance of high and low risk group in validation set. (**D**) ROC analysis of prognostic model at 1, 3, 5 year in validation set. (**E**) Kaplan-Meier survival analysis revealing the prognostic significance of high and low risk group in external validation set. (**F**) ROC analysis of prognostic model at 1, 3, 5 year in external validation set.

Prior findings revealed significant differences in biological processes and prognosis between the C1 and C2 subtypes. Based on these findings, we proceeded to develop a survival prediction model. Using the CGGA database as the training set, we first performed univariate survival analysis on the differentially expressed genes identified from the C1 and C2 subtypes. Prognosis-related genes were further refined by LASSO regression, ultimately yielding a 13-gene prognostic signature. Multivariate Cox regression was then applied to establish the model formula, Risk Score = −0.05600509 ∗ SPHKAP − 0.012254248 ∗ CSDC2 − 0.03740532 ∗ CALN1 − 0.002812162 ∗ ASIC4 − 0.004102312 ∗ SLC25A48 − 0.03367478 ∗ DGKB + 0.0444537789993795 ∗ OTP + 0.0251888002037989 ∗ SPATA6 + 0.00139784395696491 ∗ IGFBP5 + 0.0013710784782987 ∗ MAOB + 0.0135107786154736 ∗ HOXD9 + 0.0134246649058088 ∗ MYBL2 + 0.0166909013178275 ∗ MEOX2 (Supplementary Fig. S6A). Within this signature, HOXD9, MYBL2, SPATA6, MAOB, IGFBP5, OTP, and MEOX2 were up-regulated in the high-risk group, whereas DGKB, SPHKAP, CALN1, ASIC4, CSDC2, and SLC25A48 were up-regulated in the low-risk group (Supplementary Fig. S6B). A higher risk score correlated with a lower proportion of surviving patients (Supplementary Fig. S6C). Multivariate Cox regression analysis incorporating IDH mutation status, tumor grade, and other clinical parameters demonstrated that the risk score is an independent prognostic factor (Supplementary Fig. S6D). In the CGGA training cohort, we confirmed that high-risk patients exhibited significantly poorer survival outcomes. Time-dependent ROC analysis demonstrated high sensitivity and specificity of the model, with areas under the curve (AUC) of 0.80, 0.86, and 0.86 at 1, 3, and 5 years, respectively (Fig. 6A,B). When validated in the TCGA dataset, consistent results were obtained: high-risk patients had worse prognosis, with AUC of 0.69, 0.77, and 0.72 at 1, 3, and 5 years, respectively (Fig. 6C,D). Furthermore, an external validation using a GEO dataset confirmed the robust predictive performance of the model (Fig. 6E,F).

### 3.7 Inhibition of IGFBP2 Enhances Tumor Response to Anti-PD-1 Therapy

**Figure 7:**
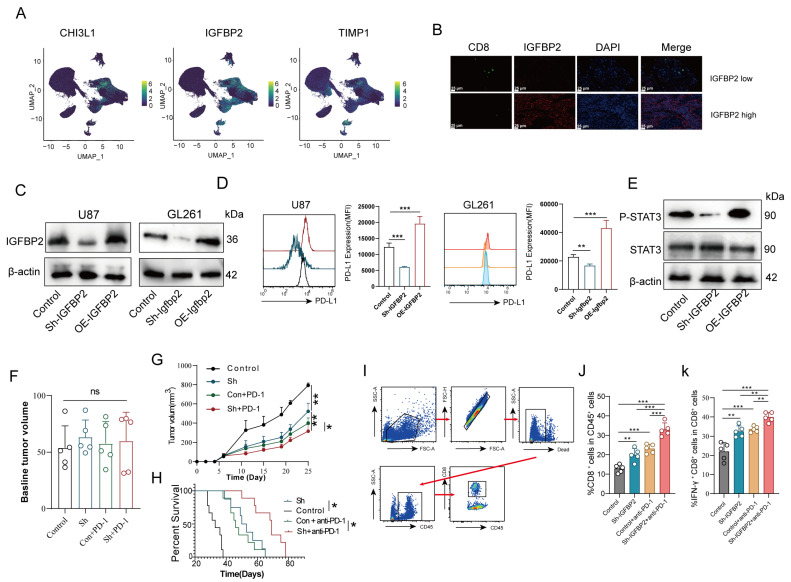
Inhibition of Insulin-Like Growth Factor Binding Protein 2 (IGFBP2) promotes anti-Programmed Cell Death Protein 1 (PD-1) treatment. (**A**) Uniform Manifold Approximation and Projection (UMAP) plot showing top3 Differentially Expressed Genes (DEGs) between C1 and C2. (**B**) Multiplex immunofluorescence reveals the expression of cluster of differentiation (CD)8 and IGFBP2 in glioma tissues. (**C**) Western blot showing the expression of IGFBP2 in the knockdown, overexpressed group and the control group. (**D**) Flow cytometry shows representative plots of PD-L1 expression on the cell surface, with statistical plots on the left. (**E**) Western blot showing the expression of Signal Transducer and Activator of Transcription 3 (STAT3) and phosphorylated(p)-STAT3 in the knockdown, overexpressed group and the control group. (**F**) Box plots showing the baseline tumor volumes across the four groups. (**G**) The tumor growth curves of mice from the four treatment groups. (**H**) The survival time of mice in the four treatment groups. (**I**) Flowchart for analyzing the proportion of CD8^+^ T cells in mouse tissues by flow cytometry. (**J**) Box plot showing the proportion of CD8^+^ T cells. (**K**) Box plot showing the proportion of interferon-gamma (IFN-γ) ^+^CD8^+^ T cells. *p < 0.05, **p < 0.01, ***p < 0.001. ns: not significant.

To further investigate the mechanisms by which the C1 and C2 subtypes affect immunity and prognosis, we first analyzed the expression of DEGs between C1 and C2 subtypes. We found that the IGFBP2 gene was predominantly expressed in tumor cells, suggesting its potential as a therapeutic target (Fig. 7A). Additionally, analysis of patient specimens revealed that high expression of IGFBP2 was negatively correlated with the presence of CD8^+^ T cells (Fig. 7B). Subsequently, we validated the role of IGFBP2 through both *in vitro* and *in vivo* experiments. We performed IGFBP2 knockdown and overexpression in both human and mouse cell lines, and the efficiency was confirmed by Western blotting. The results showed that IGFBP2 knockdown significantly reduced its expression, whereas overexpression markedly increased its expression (Fig. 7C). Previous studies have shown that IGFBP2 can regulate immune-related pathways, such as PD-L1 [41,42]. Therefore, we examined PD-L1 expression on tumor cells. We found that IGFBP2 knockdown significantly reduced PD-L1 levels, whereas IGFBP2 overexpression markedly increased its expression. (Fig. 7D). Further mechanistic analyses revealed that IGFBP2 knockdown inhibited STAT3 phosphorylation, whereas its overexpression promoted STAT3 phosphorylation (Fig. 7E). *In vivo* experiments demonstrated that tumors in the knockdown group grew significantly slower than those in the control group. Moreover, treatment with an anti-PD-1 antibody further inhibited tumor growth (Fig. 7F,G). Survival analysis further indicated that IGFBP2 knockdown prolonged mouse survival, and this effect was enhanced when combined with anti-PD-1 therapy (Fig. 7H). We then analyzed the phenotype and function of T cells in tumor tissues. In the IGFBP2 knockdown group, CD8^+^ T cells showed a higher infiltration (Fig. 7I,J), along with enhanced secretion of IFN-γ. Moreover, combination therapy with a PD-1 monoclonal antibody significantly enhanced the functionality of tumor-infiltrating CD8^+^ T cells (Fig. 7K).

## 4 Discussion

Glioma is one of the brain tumors with the most lethal rate. Due to its tumor heterogeneity and complex microenvironment, current targeted therapies and immunotherapies achieved limited clinical benefits. Tumors are prone to recurrence, and patients generally experience short overall survival [43]. Our research employed single-cell sequencing techniques to analyze data obtained from primary and recurrent tumor samples in both LGG and GBM. This approach allowed us to systematically dissect the immunosuppressive conditions within glioma microenvironments at cellular resolution.

We identified a total of six cell clusters, consistent with findings from previously previous reports [39,40]. Among these clusters, we first analyzed the phenotype and function of T cells. T cells are the primary effectors to immune checkpoint inhibitor therapy and serve as key effectors in mediating immune responses in the body [44]. We observed that these cells highly expressed immunosuppressive molecules such as PDCD1 (encoding PD-1), HAVCR2 (encoding Tim-3), and CTLA-4 in GBM and recurrent tumor tissues. Cells expressing these molecules are currently represent exhausted T cells [45], which are characterized by reduced cytokine secretion, diminished proliferative capacity, and increased apoptosis [46]. Recent studies have further revealed that in LGG, a subset of exhausted T cells can secrete paracrine factors essential for low-grade glioma growth, thereby promoting tumor progression [47]. Meanwhile, protein-based spatial subtyping further indicated that patients with glioma enriched in this exhausted T-cell subpopulation exhibited poorer survival [48]. In this study, we found that the high expression of this cell population was also correlated with poor patient prognosis. Consistent with this observation, in GBM and recurrent tumors, we identified activation of the HIF-1α pathway in T cells. This pathway is not only critical for sustaining tumor progression but also a key factor driving T cells toward terminal exhaustion [49,50]. These results indicate that T-cell function is suppressed in more malignant tumor tissues.

In glioma, myeloid cells represent the most abundant non-tumor cellular component [51], which aligns with these findings: myeloid cells represent the largest proportion among non-tumor cells in glioma tissue. Furthermore, we observed that these myeloid cells exhibit an immunosuppressive phenotype in GBM and recurrent tumors, characterized by high expression of molecules such as IL-6, IL-10, and MIF. Numerous studies have demonstrated that myeloid cells within the glioma microenvironment contribute to the phenotypic transition of glioma cells and suppress T-cell function [52,53]. Among myeloid cells, myeloid-derived suppressor cells (MDSCs) and tumor-associated macrophages (TAMs) are the major immunosuppressive subsets [54]. Moreover, they display high phenotypic heterogeneity within the microenvironment [55,56]. In IDH-wildtype GBM, two distinct MDSC populations are present: early MDSC progenitors and monocytic MDSCs (M-MDSCs). Early MDSCs (E-MDSCs) co-localize with metabolically active stem-like tumor cells in the perinecrotic pseudopalisading regions, where they promote tumor cell proliferation [40]. In this study, we also identified a subset of S100A8^+^ neutrophils characterized by the activation of pathways such as EGFR and PI3K, which showed a negative correlation with patient prognosis. This finding suggests that these cells may represent a population of tumor-associated neutrophils (TANs) [57].

Tumor heterogeneity remains a major obstacle in current treatment strategies. Although molecular classifications have been established for gliomas, these classifications are primarily based on traditional bulk sequencing data [12]. Our findings reveal 12 distinct subpopulations of glioma cells, which allowed us to stratify into two major glioma subtypes. A distinct variant exhibits heightened immune cell presence, particularly T lymphocytes and B lymphocytes, while simultaneously containing immunosuppressive cells like regulatory T cells (Tregs) and myeloid-derived suppressor cells (MDSCs). Consistent with this, multi-omics-based classification of gliomas has identified four subtypes, one of which is the immune/mesenchymal-enriched subtype. This subtype exhibits abundant immune infiltration; however, the T cells predominantly display an exhausted phenotype, leading to a negative correlation with prognosis [48]. These results also indicate that despite the high immune cell infiltration, patients with this subtype have a poorer prognosis. Our previous study based on metabolic profiling similarly demonstrated that gliomas with substantial immune infiltration are associated with worse outcomes [58]. Beyond classifications based on molecular or single-cell data, imaging-based analysis also serves as an important tool for glioma stratification [59].

A pivotal finding of this study suggests that IGFBP2 may serve as a potential therapeutic target [60]. In the present study, the molecular subtyping model and the selection of IGFBP2 were based on distinct analytical strategies. The molecular subtyping model was constructed using marker genes derived from glioma cell subpopulations identified through single-cell analysis, aiming to capture the intrinsic heterogeneity of tumor cell states. In contrast, IGFBP2 was identified through differential expression analysis between the C1 and C2 subtypes and was found to be significantly associated with poor prognosis. Importantly, IGFBP2 was not included as a variable in the construction of the prognostic model, nor was it selected through LASSO regression or evaluated as an independent factor in multivariate Cox regression within the model framework. Instead, it was investigated as a representative gene with potential biological relevance among subtype-associated differentially expressed genes. Therefore, the current findings support a correlation between IGFBP2 expression and glioma subtype characteristics and clinical outcomes, rather than establishing IGFBP2 as a core driver of the C2 subtype or a determinant variable within the predictive model. Future studies integrating larger cohorts and mechanistic experiments will be required to further clarify its causal role.

Mounting evidence have reported that IGFBP2 promotes tumor progression. In gliomas, IGFBP2 induces the polarization of M2 macrophages, thereby accelerates tumor growth [61]. Furthermore, IGFBP2-enriched microparticles enhance tumor stemness and confer resistance to radiotherapy [62]. In non-small cell lung cancer, IGFBP2 promotes gefitinib resistance through the STAT3/CXCL1 pathway [63]. In this study, we observed that IGFBP2 is predominantly highly expressed in immune-enriched subtypes and is associated with poor patient prognosis, suggesting that it may influence patient survival by modulating immune responses. Further investigation revealed that knockdown of IGFBP2 significantly downregulates PD-L1 expression on tumor cell surfaces. In melanoma, IGFBP2 has been shown to regulate PD-L1 expression via the EGFR-STAT3 pathway [64]. Additionally, high baseline tumor burden-associated macrophages contribute to an immunosuppressive microenvironment through the IGFBP2-PD-L1 axis [41]. Notably, in gliomas, we found that suppressing IGFBP2 expression markedly inhibits PD-L1 expression and enhances the efficacy of anti-PD-1 monoclonal antibody therapy.

Several strategies targeting IGFBP2 to suppress tumors have shown promising antitumor effects. For example, in breast cancer, antisense oligonucleotides have used to inhibit IGFBP2 mRNA translation, thereby inhibiting its expression level and suppressing tumor cell growth [65]. In glioma, another study has shown that neutralizing antibodies or single-chain monoclonal antibodies can block IGFBP2-mediated activation of downstream pro-tumor pathways, thereby inhibiting tumor invasion [66,67]. Moreover, peptide fragments derived from IGFBP2 are recognizable by the immune system, and vaccines designed on this basis can activate immune responses to suppress tumor growth [68]. The high-precision 3D structure of IGFBP2 predicted by AlphaFold3 can be used for computational simulation and high-throughput virtual screening [69]. This approach will allow systematic identification of small-molecule candidates from large compound libraries that specifically target the active sites of IGFBP2, thereby enabling precise targeting of this protein.

However, translating these IGFBP2-targeting strategies into clinical practice for GBM faces substantial challenges. GBM is characterized by its location within the central nervous system (CNS), where the blood-brain barrier (BBB) severely limits drug delivery to brain tumors [70]. Currently, several strategies have been proposed to target the BBB. These include the chemical modification of drugs, or the use of nanoparticles to encapsulate drugs and modify their surfaces to mimic biomolecules, which can enhance BBB penetration and targeted delivery to glioma [71]. Regarding potential adverse effects, targeting IGFBP2 may lead to off-target toxicity due to the widespread expression of IGFBP2 in normal tissues and its multifunctional role in physiological processes [72]. To address these challenges, future research should focus on optimizing drug delivery systems to enhance BBB penetration and tumor-specific accumulation of IGFBP2-targeting agents.

This study has several limitations. First, the integrated datasets were originated from different studies, which may introduce batch effects and technical variability despite the application of integration methods. Second, the unequal sample sizes among clinical subgroups may affect the representation of rare cell populations. Third, the subcutaneous xenograft model used in this study does not fully recapitulate the intracranial glioma microenvironment. In particular, the brain-specific anatomical structure, blood-brain barrier, resident glial populations, and unique immune contexture does not adequately represented in subcutaneous tumors.

## 5 Conclusion

This investigation focused on examining the cellular makeup of the tumor microenvironment and assessing tumor cell diversity in both low-grade and high-grade gliomas through single-cell analysis. The results demonstrated that within aggressive and recurring tumor samples, T lymphocytes showed signs of functional depletion, whereas myeloid-derived cells adopted an immunosuppressive profile. Through evaluation of glioma cell variability, we successfully categorized the malignant cells into two distinct subgroups. One subtype was enriched with immune cells, with infiltrating MDSCs and Tregs negatively correlated with patient prognosis. Using the differentially expressed genes between the two subtypes, we constructed a survival prediction model. We discovered that IGFBP2 may be associated with poor prognosis, potentially through PD-L1 regulation. Overall, this article provides an in-depth exploration of tumor tissue heterogeneity and suggests IGFBP2 as a potential therapeutic target.

## Data Availability

The data that support the findings of this study are available from the corresponding author upon reasonable request.

## Supplementary Materials

The supplementary material is available online at https://www.techscience.com/doi/10.32604/or.2026.079221/s1.

## References

[1] Lamba N , Wen PY , Aizer AA . Epidemiology of brain metastases and leptomeningeal disease. Neuro Oncol. 2021; 23( 9): 1447– 56. doi:10.1093/neuonc/noab101. 33908612 PMC8408881

[2] Ostrom QT , Price M , Neff C , Cioffi G , Waite KA , Kruchko C , et al. CBTRUS statistical report: Primary brain and other central nervous system tumors diagnosed in the United States in 2016–2020. Neuro Oncol. 2023; 25( 12 Suppl 2): iv1– 99. doi:10.1093/neuonc/noad149. 37793125 PMC10550277

[3] Sánchez ML , Mangas A , Coveñas R . Glioma and peptidergic systems: Oncogenic and anticancer peptides. Int J Mol Sci. 2024; 25( 14): 7990. doi:10.3390/ijms25147990. 39063232 PMC11277022

[4] Chen R , Smith-Cohn M , Cohen AL , Colman H . Glioma subclassifications and their clinical significance. Neurotherapeutics. 2017; 14( 2): 284– 97. doi:10.1007/s13311-017-0519-x. 28281173 PMC5398991

[5] Weller M , Wen PY , Chang SM , Dirven L , Lim M , Monje M , et al. Glioma. Nat Rev Dis Primers. 2024; 10( 1): 33. doi:10.1038/s41572-024-00516-y. 38724526

[6] Ohgaki H , Kleihues P . Population-based studies on incidence, survival rates, and genetic alterations in astrocytic and oligodendroglial gliomas. J Neuropathol Exp Neurol. 2005; 64( 6): 479– 89. doi:10.1093/jnen/64.6.479. 15977639

[7] Bleeker FE , Molenaar RJ , Leenstra S . Recent advances in the molecular understanding of glioblastoma. J Neuro Oncol. 2012; 108( 1): 11– 27. doi:10.1007/s11060-011-0793-0. PMC3337398 22270850

[8] Yasinjan F , Xing Y , Geng H , Guo R , Yang L , Liu Z , et al. Immunotherapy: A promising approach for glioma treatment. Front Immunol. 2023; 14: 1255611. doi:10.3389/fimmu.2023.1255611. 37744349 PMC10512462

[9] Yang K , Wu Z , Zhang H , Zhang N , Wu W , Wang Z , et al. Glioma targeted therapy: Insight into future of molecular approaches. Mol Cancer. 2022; 21( 1): 39. doi:10.1186/s12943-022-01513-z. 35135556 PMC8822752

[10] Saito R . Chemotherapy for glioma. No Shinkei Geka Neurol Surg. 2021; 49( 3): 588– 96. 10.11477/mf.1436204432 34092564

[11] Louis DN , Perry A , Reifenberger G , Von Deimling A , Figarella-Branger D , Cavenee WK , et al. The 2016 World Health Organization classification of tumors of the central nervous system: A summary. Acta Neuropathol. 2016; 131( 6): 803– 20. doi:10.1007/s00401-016-1545-1. 27157931

[12] Louis DN , Perry A , Wesseling P , Brat DJ , Cree IA , Figarella-Branger D , et al. The 2021 WHO classification of tumors of the central nervous system: A summary. Neuro Oncol. 2021; 23( 8): 1231– 51. doi:10.1093/neuonc/noab106. 34185076 PMC8328013

[13] van der Voort SR , Incekara F , Wijnenga MM , Kapsas G , Gahrmann R , Schouten JW , et al. Combined molecular subtyping, grading, and segmentation of glioma using multi-task deep learning. Neuro Oncol. 2023; 25( 2): 279– 89. doi:10.1093/neuonc/noac166. 35788352 PMC9925710

[14] Auffret L , Ajlil Y , Tauziède-Espariat A , Kergrohen T , Puiseux C , Riffaud L , et al. A new subtype of diffuse midline glioma, H3 K27 and BRAF/FGFR1 co-altered: A clinico-radiological and histomolecular characterisation. Acta Neuropathol. 2024; 147( 1): 2. doi:10.1007/s00401-023-02651-4. PMC10709479 38066305

[15] Muench A , Teichmann D , Spille D , Kuzman P , Perez E , May SA , et al. A novel type of IDH-wildtype glioma characterized by gliomatosis cerebri-like growth pattern, TERT promoter mutation, and distinct epigenetic profile. Am J Surg Pathol. 2023; 47( 12): 1364– 75. doi:10.1097/PAS.0000000000002118. 37737691

[16] Eisenbarth D , Wang YA . Glioblastoma heterogeneity at single cell resolution. Oncogene. 2023; 42( 27): 2155– 65. doi:10.1038/s41388-023-02738-y. 37277603 PMC10913075

[17] Jovic D , Liang X , Zeng H , Lin L , Xu F , Luo Y . Single-cell RNA sequencing technologies and applications: A brief overview. Clin Transl Med. 2022; 12( 3): e694. doi:10.1002/ctm2.694. 35352511 PMC8964935

[18] Boxer E , Feigin N , Tschernichovsky R , Darnell NG , Greenwald AR , Hoefflin R , et al. Emerging clinical applications of single-cell RNA sequencing in oncology. Nat Rev Clin Oncol. 2025; 22( 5): 315– 26. doi:10.1038/s41571-025-01003-3. 40021788

[19] Phillips HS , Kharbanda S , Chen R , Forrest WF , Soriano RH , Wu TD , et al. Molecular subclasses of high-grade glioma predict prognosis, delineate a pattern of disease progression, and resemble stages in neurogenesis. Cancer Cell. 2006; 9( 3): 157– 73. doi:10.1016/j.ccr.2006.02.019. 16530701

[20] Verhaak RG , Hoadley KA , Purdom E , Wang V , Qi Y , Wilkerson MD , et al. Integrated genomic analysis identifies clinically relevant subtypes of glioblastoma characterized by abnormalities in PDGFRA, IDH1, EGFR, and NF1. Cancer Cell. 2010; 17( 1): 98– 110. doi:10.1016/j.ccr.2009.12.020. 20129251 PMC2818769

[21] Wang Q , Hu B , Hu X , Kim H , Squatrito M , Scarpace L , et al. Tumor evolution of glioma-intrinsic gene expression subtypes associates with immunological changes in the microenvironment. Cancer Cell. 2017; 32( 1): 42– 56. doi:10.1016/j.ccell.2017.06.003. 28697342 PMC5599156

[22] Patel AP , Tirosh I , Trombetta JJ , Shalek AK , Gillespie SM , Wakimoto H , et al. Single-cell RNA-seq highlights intratumoral heterogeneity in primary glioblastoma. Science. 2014; 344( 6190): 1396– 401. doi:10.1126/science.1254257. 24925914 PMC4123637

[23] Neftel C , Laffy J , Filbin MG , Hara T , Shore ME , Rahme GJ , et al. An integrative model of cellular states, plasticity, and genetics for glioblastoma. Cell. 2019; 178( 4): 835– 49. doi:10.1016/j.cell.2019.06.024. 31327527 PMC6703186

[24] Liu I , Jiang L , Samuelsson ER , Marco Salas S , Beck A , Hack OA , et al. The landscape of tumor cell states and spatial organization in H3-K27M mutant diffuse midline glioma across age and location. Nat Genet. 2022; 54( 12): 1881– 94. doi:10.1038/s41588-022-01236-3. 36471067 PMC9729116

[25] Stupp R , Mason WP , Van Den Bent MJ , Weller M , Fisher B , Taphoorn MJ , et al. Radiotherapy plus concomitant and adjuvant temozolomide for glioblastoma. N Engl J Med. 2005; 352( 10): 987– 96. doi:10.1056/NEJMoa043330. 15758009

[26] Nicholson JG , Fine HA . Diffuse glioma heterogeneity and its therapeutic implications. Cancer Discov. 2021; 11( 3): 575– 90. doi:10.1158/2159-8290.CD-20-1474. 33558264

[27] Friedman HS , Prados MD , Wen PY , Mikkelsen T , Schiff D , Abrey LE , et al. Bevacizumab alone and in combination with irinotecan in recurrent glioblastoma. J Clin Oncol. 2009; 27( 28): 4733– 40. doi:10.1200/JCO.2008.19.8721. 19720927

[28] Cohen MH , Shen YL , Keegan P , Pazdur R . FDA drug approval summary: Bevacizumab (Avastin^®^) as treatment of recurrent glioblastoma multiforme. Oncologist. 2009; 14( 11): 1131– 8. doi:10.1634/theoncologist.2009-0121. 19897538

[29] Kirson ED , Gurvich Z , Schneiderman R , Dekel E , Itzhaki A , Wasserman Y , et al. Disruption of cancer cell replication by alternating electric fields. Cancer Res. 2004; 64( 9): 3288– 95. doi:10.1158/0008-5472.CAN-04-0083. 15126372

[30] Stupp R , Taillibert S , Kanner AA , Kesari S , Steinberg DM , Toms SA , et al. Maintenance therapy with tumor-treating fields plus temozolomide vs. temozolomide alone for glioblastoma: A randomized clinical trial. JAMA. 2015; 314( 23): 2535– 43. doi:10.1001/jama.2015.16669. 26670971

[31] Xu S , Tang L , Li X , Fan F , Liu Z . Immunotherapy for glioma: Current management and future application. Cancer Lett. 2020; 476: 1– 2. doi:10.1016/j.canlet.2020.02.002. 32044356

[32] de Melo SM , da Silva ME , Torloni MR , Riera R , De Cicco K , Latorraca CO , et al. Anti-PD-1 and anti-PD-L1 antibodies for glioma. Cochrane Database Syst Rev. 2025; 1( 1): cd012532. doi:10.1002/14651858.CD012532.pub2. 39777725 PMC11707826

[33] Jiacheng D , Jiayue C , Ying G , Shaohua W , Wenhui L , Xinyu H . Research progress and challenges of the PD-1/PD-L1 axis in gliomas. Cell Biosci. 2024; 14( 1): 123. doi:10.1186/s13578-024-01305-6. 39334448 PMC11437992

[34] Cloughesy TF , Mochizuki AY , Orpilla JR , Hugo W , Lee AH , Davidson TB , et al. Neoadjuvant anti-PD-1 immunotherapy promotes a survival benefit with intratumoral and systemic immune responses in recurrent glioblastoma. Nat Med. 2019; 25( 3): 477– 86. doi:10.1038/s41591-018-0337-7. 30742122 PMC6408961

[35] Omuro A , Vlahovic G , Lim M , Sahebjam S , Baehring J , Cloughesy T , et al. Nivolumab with or without ipilimumab in patients with recurrent glioblastoma: Results from exploratory phase I cohorts of CheckMate 143. Neuro-Oncology. 2018; 20( 5): 674– 86. doi:10.1093/neuonc/nox208. 29106665 PMC5892140

[36] Komohara Y , Ohnishi K , Kuratsu J , Takeya M . Possible involvement of the M2 anti-inflammatory macrophage phenotype in growth of human gliomas. J Pathol. 2008; 216( 1): 15– 24. doi:10.1002/path.2370. 18553315

[37] Crane CA , Ahn BJ , Han SJ , Parsa AT . Soluble factors secreted by glioblastoma cell lines facilitate recruitment, survival, and expansion of regulatory T cells: Implications for immunotherapy. Neuro-Oncology. 2012; 14( 5): 584– 95. doi:10.1093/neuonc/nos014. 22406925 PMC3337302

[38] Schmassmann P , Roux J , Dettling S , Hogan S , Shekarian T , Martins TA , et al. Single-cell characterization of human GBM reveals regional differences in tumor-infiltrating leukocyte activation. eLife. 2023; 12: RP92678. doi:10.7554/eLife.92678. 38127790 PMC10735226

[39] Abdelfattah N , Kumar P , Wang C , Leu JS , Flynn WF , Gao R , et al. Single-cell analysis of human glioma and immune cells identifies S100A4 as an immunotherapy target. Nat Commun. 2022; 13( 1): 767. doi:10.1038/s41467-022-28372-y. 35140215 PMC8828877

[40] Wang L , Jung J , Babikir H , Shamardani K , Jain S , Feng X , et al. A single-cell atlas of glioblastoma evolution under therapy reveals cell-intrinsic and cell-extrinsic therapeutic targets. Nat Cancer. 2022; 3( 12): 1534– 52. doi:10.1038/s43018-022-00475-x. 36539501 PMC9767870

[41] Wen Z , Sun H , Zhang Z , Zheng Y , Zheng S , Bin J , et al. High baseline tumor burden-associated macrophages promote an immunosuppressive microenvironment and reduce the efficacy of immune checkpoint inhibitors through the IGFBP2-STAT3-PD-L1 pathway. Cancer Commun. 2023; 43( 5): 562– 81. doi:10.1002/cac2.12420. PMC10174084 37031362

[42] Narote S , Desai SA , Patel VP , Deshmukh R , Raut N , Dapse S . Identification of new immune target and signaling for cancer immunotherapy. Cancer Genet. 2025; 294: 57– 75. doi:10.1016/j.cancergen.2025.03.004. 40154216

[43] Gritsch S , Batchelor TT , Gonzalez Castro LN . Diagnostic, therapeutic, and prognostic implications of the 2021 World Health Organization classification of tumors of the central nervous system. Cancer. 2022; 128( 1): 47– 58. doi:10.1002/cncr.33918. 34633681

[44] O’Donnell JS , Teng MW , Smyth MJ . Cancer immunoediting and resistance to T cell-based immunotherapy. Nat Rev Clin Oncol. 2019; 16( 3): 151– 67. doi:10.1038/s41571-018-0142-8. 30523282

[45] Baessler A , Vignali DA . T cell exhaustion. Annu Rev Immunol. 2024; 42( 1): 179– 206. doi:10.1146/annurev-immunol-090222-110914. 38166256

[46] Brunell AE , Lahesmaa R , Autio A , Thotakura AK . Exhausted T cells hijacking the cancer-immunity cycle: Assets and liabilities. Front Immunol. 2023; 14: 1151632. doi:10.3389/fimmu.2023.1151632. 37122741 PMC10140554

[47] Barakat R , Chatterjee J , Mu R , Qi X , Gu X , Smirnov I , et al. Human single cell RNA-sequencing reveals a targetable CD8^+^ exhausted T cell population that maintains mouse low-grade glioma growth. Nat Commun. 2024; 15( 1): 10312. doi:10.1038/s41467-024-54569-4. 39609412 PMC11605098

[48] Tang J , Fan W , Ruan Y , Liu X , Qiu F , Feng J , et al. Protein-based classification reveals an immune-hot subtype in IDH mutant astrocytoma with worse prognosis. Cancer Cell. 2025; 43( 11): 2136– 55. doi:10.1016/j.ccell.2025.08.006. 40939590

[49] Paredes F , Williams HC , San Martin A . Metabolic adaptation in hypoxia and cancer. Cancer Lett. 2021; 502: 133– 42. doi:10.1016/j.canlet.2020.12.020. 33444690 PMC8158653

[50] Wu H , Zhao X , Hochrein SM , Eckstein M , Gubert GF , Knöpper K , et al. Mitochondrial dysfunction promotes the transition of precursor to terminally exhausted T cells through HIF-1α-mediated glycolytic reprogramming. Nat Commun. 2023; 14( 1): 6858. doi:10.1038/s41467-023-42634-3. 37891230 PMC10611730

[51] Klemm F , Maas RR , Bowman RL , Kornete M , Soukup K , Nassiri S , et al. Interrogation of the microenvironmental landscape in brain tumors reveals disease-specific alterations of immune cells. Cell. 2020; 181( 7): 1643– 60. doi:10.1016/j.cell.2020.05.007. 32470396 PMC8558904

[52] Hara T , Chanoch-Myers R , Mathewson ND , Myskiw C , Atta L , Bussema L , et al. Interactions between cancer cells and immune cells drive transitions to mesenchymal-like states in glioblastoma. Cancer Cell. 2021; 39( 6): 779– 92. doi:10.1016/j.ccell.2021.05.002. 34087162 PMC8366750

[53] Mathewson ND , Ashenberg O , Tirosh I , Gritsch S , Perez EM , Marx S , et al. Inhibitory CD161 receptor identified in glioma-infiltrating T cells by single-cell analysis. Cell. 2021; 184( 5): 1281– 98. doi:10.1016/j.cell.2021.01.022. 33592174 PMC7935772

[54] Mirghorbani M , Van Gool S , Rezaei N . Myeloid-derived suppressor cells in glioma. Expert Rev Neurother. 2013; 13( 12): 1395– 406. doi:10.1586/14737175.2013.857603. 24215283

[55] Jackson C , Cherry C , Bom S , Dykema AG , Wang R , Thompson E , et al. Distinct myeloid-derived suppressor cell populations in human glioblastoma. Science. 2025; 387( 6731): eabm5214. doi:10.1126/science.abm5214. 39818911 PMC12836367

[56] Ochocka N , Segit P , Walentynowicz KA , Wojnicki K , Cyranowski S , Swatler J , et al. Single-cell RNA sequencing reveals functional heterogeneity of glioma-associated brain macrophages. Nat Commun. 2021; 12( 1): 1151. doi:10.1038/s41467-021-21407-w. 33608526 PMC7895824

[57] Que H , Fu Q , Lan T , Tian X , Wei X . Tumor-associated neutrophils and neutrophil-targeted cancer therapies. Biochim Et Biophys Acta (BBA) Rev Cancer. 2022; 1877( 5): 188762. doi:10.1016/j.bbcan.2022.188762. 35853517

[58] Kang J , Xu Y , Zhao Q , Wang Y , He Z , Xu X . Metabolic profiling of glioblastoma and identification of G0S2 as a metabolic target. Front Oncol. 2025; 15: 1572040. doi:10.3389/fonc.2025.1572040. 40519301 PMC12162274

[59] Ohmura K , Kumagai N , Kumagai M , Ikegame Y , Shinoda J , Yano H , et al. Combining methionine-PET and MRI fluid-attenuated inversion-recovery mismatch to determine glioma molecular subtype. J Neuroimaging. 2023; 33( 4): 652– 60. doi:10.1111/jon.13114. 37158779

[60] Liu H , Tang T . A bioinformatic study of IGFBPs in glioma regarding their diagnostic, prognostic, and therapeutic prediction value. Am J Transl Res. 2023; 15( 3): 2140. 37056850 PMC10086936

[61] Zhang X , Sun X , Guo C , Li J , Liang G . Cancer-associated fibroblast-associated gene IGFBP2 promotes glioma progression through induction of M2 macrophage polarization. Am J Physiol Cell Physiol. 2024; 326( 1): C252– 68. doi:10.1152/ajpcell.00234.2023. 37982173

[62] Yang Y , Sun T , Xue X , Tan H , Li Y , Yang W . HIG-2 promotes glioma stemness and radioresistance mediated by IGFBP2-rich microparticles in hypoxia. Apoptosis. 2025; 30( 1): 297– 319. doi:10.1007/s10495-024-02045-1. 39633113

[63] Lu H , Ai J , Zheng Y , Zhou W , Zhang L , Zhu J , et al. IGFBP2/ITGA5 promotes gefitinib resistance via activating STAT3/CXCL1 axis in non-small cell lung cancer. Cell Death Dis. 2024; 15( 6): 447. doi:10.1038/s41419-020-2650-6. 38918360 PMC11199710

[64] Li T , Zhang C , Zhao G , Zhang X , Hao M , Hassan S , et al. IGFBP2 regulates PD-L1 expression by activating the EGFR-STAT3 signaling pathway in malignant melanoma. Cancer Lett. 2020; 477: 19– 30. doi:10.1016/j.canlet.2020.02.036. 32120023 PMC7816098

[65] So AI , Levitt RJ , Eigl B , Fazli L , Muramaki M , Leung S , et al. Insulin-like growth factor binding protein-2 is a novel therapeutic target associated with breast cancer. Clin Cancer Res. 2008; 14( 21): 6944– 54. doi:10.1158/1078-0432.CCR-08-0408. 18980989

[66] Phillips LM , Zhou X , Cogdell DE , Chua CY , Huisinga A , R Hess K , et al. Glioma progression is mediated by an addiction to aberrant IGFBP2 expression and can be blocked using anti-IGFBP2 strategies. J Pathol. 2016; 239( 3): 355– 64. doi:10.1002/path.4734. 27125842 PMC4915980

[67] Patil SS , Railkar R , Swain M , Atreya HS , Dighe RR , Kondaiah P . Novel anti IGFBP2 single chain variable fragment inhibits glioma cell migration and invasion. J Neuro Oncol. 2015; 123( 2): 225– 35. doi:10.1007/s11060-015-1800-7. 25944386

[68] Park KH , Gad E , Goodell V , Dang Y , Wild T , Higgins D , et al. Insulin-like growth factor–binding protein-2 is a target for the immunomodulation of breast cancer. Cancer Res. 2008; 68( 20): 8400– 9. doi:10.1158/0008-5472.CAN-07-5891. 18922913 PMC2596961

[69] Krokidis MG , Koumadorakis DE , Lazaros K , Ivantsik O , Exarchos TP , Vrahatis AG , et al. AlphaFold3: An overview of applications and performance insights. Int J Mol Sci. 2025; 26( 8): 3671. doi:10.3390/ijms26083671. 40332289 PMC12027460

[70] Wang J , Wang Z , Zhang G , Rodrigues J , Tomás H , Shi X , et al. Blood–brain barrier-crossing dendrimers for glioma theranostics. Biomater Sci. 2024; 12( 6): 1346– 56. doi:10.1039/D4BM00043A. 38362780

[71] Narsinh KH , Perez E , Haddad AF , Young JS , Savastano L , Villanueva-Meyer JE , et al. Strategies to improve drug delivery across the blood–brain barrier for glioblastoma. Curr Neurol Neurosci Rep. 2024; 24( 5): 123– 39. doi:10.1007/s11910-024-01338-x. 38578405 PMC11016125

[72] Lin YH , Wei Y , Zeng Q , Wang Y , Pagani CA , Li L , et al. IGFBP2 expressing midlobular hepatocytes preferentially contribute to liver homeostasis and regeneration. Cell Stem Cell. 2023; 30( 5): 665– 76. doi:10.1016/j.stem.2023.04.007. 37146585 PMC10580294

