# Supplementary material for: Single-Cell Sequencing Reveals the Heterogeneity of Glioma and Identifies IGFBP2 as A Potential Therapeutic Target
Source: Oncol Res. 2026 Jun 16;34(7):23. doi: 10.32604/or.2026.079221 (PMC13291989; doi:10.32604/or.2026.079221)
Supplement: Supplementary file 1 [file OncolRes-34-79221-s001.zip › TSP_OR_79221-s001.docx]

**Supplementary Figures and Legends**

**
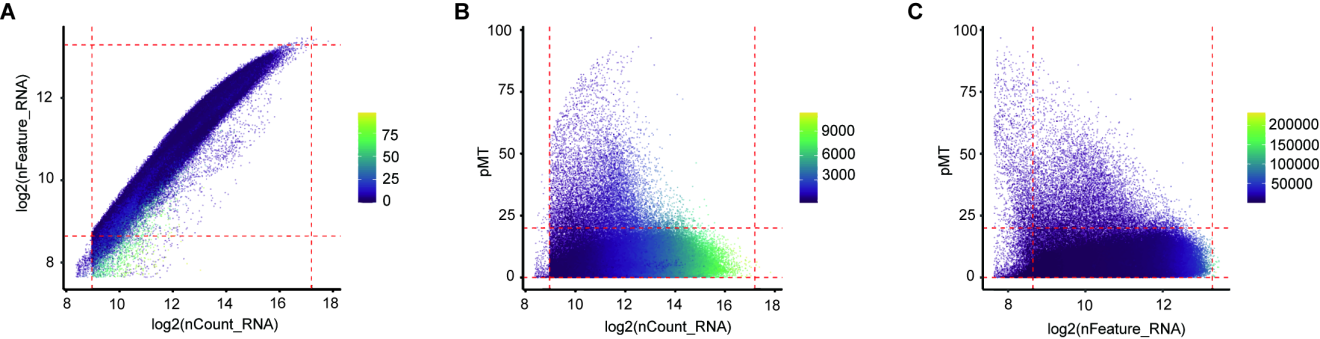
**

**Supplementary Figure S1: Quality control of the data. (A-C) Scatter plots showing the correlation between log2-transformed RNA read counts, log2-transformed number of detected RNA genes (log2(nFeature_RNA)) per cell and the proportion of mitochondrial genes.
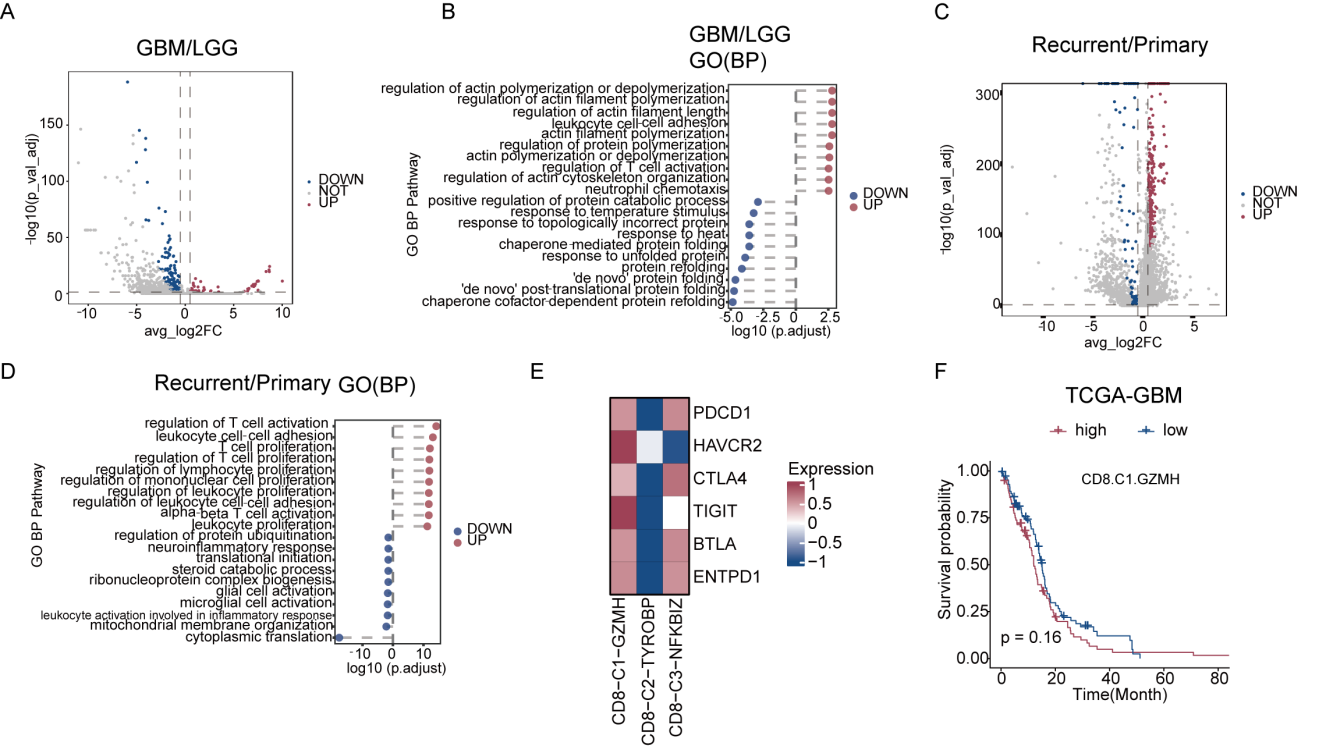
**

**Supplementary Figure S2: Functional characterization of T cell clusters. (A)The volcano plot illustrating the Differentially Expressed Genes (DEGs) in T cells between glioblastoma multiforme(GBM) and low-grade glioma (LGG). (B)Gene Ontology (GO) enrichment analysis of DEGs between GBM and LGG. (C)The volcano plot illustrating the DEGs in T cells between primary and recurrent tumor. (D)GO enrichment analysis of DEGs between primary and recurrent tumor. (E)The heatmap displaying the expression of exhaustion-related genes in the cluster of differentiation (CD8)^+^ cluster. (F)The Kaplan-Meier curve illustrating the relationship between the infiltration level of CD8-C1-Granzyme H(GZMH) and patient survival prognosis in GBM from the The Cancer Genome Atlas (TCGA) database.**

**
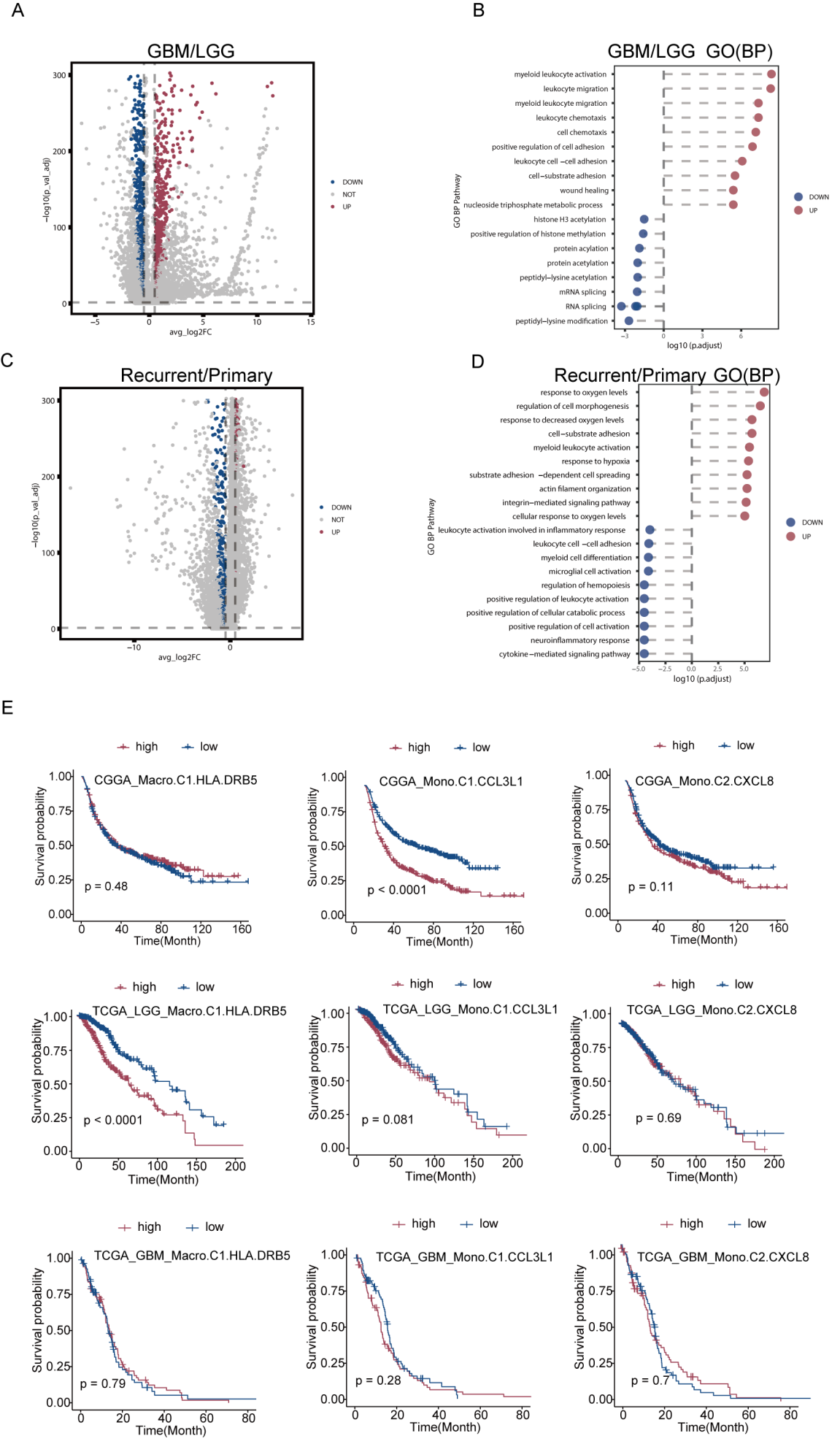
**

**Supplementary Figure S3: Characterization of myeloid cell clusters. (A)The volcano plot illustrating the Differentially Expressed Genes (DEGs) in myeloid cells between glioblastoma multiforme(GBM) and low-grade glioma (LGG). (B)Gene Ontology (GO) enrichment analysis of DEGs between GBM and LGG. (C)The volcano plot illustrating the DEGs in myeloid cells between primary and recurrent tumor. (D)GO enrichment analysis of DEGs between primary and recurrent tumor. (E)The Kaplan-Meier curve illustrating the relationship between the infiltration level of myeloid cell clusters and patient survival prognosis in The Cancer Genome Atlas (TCGA) and Chinese Glioma Genome Atlas (CGGA) database.**

**
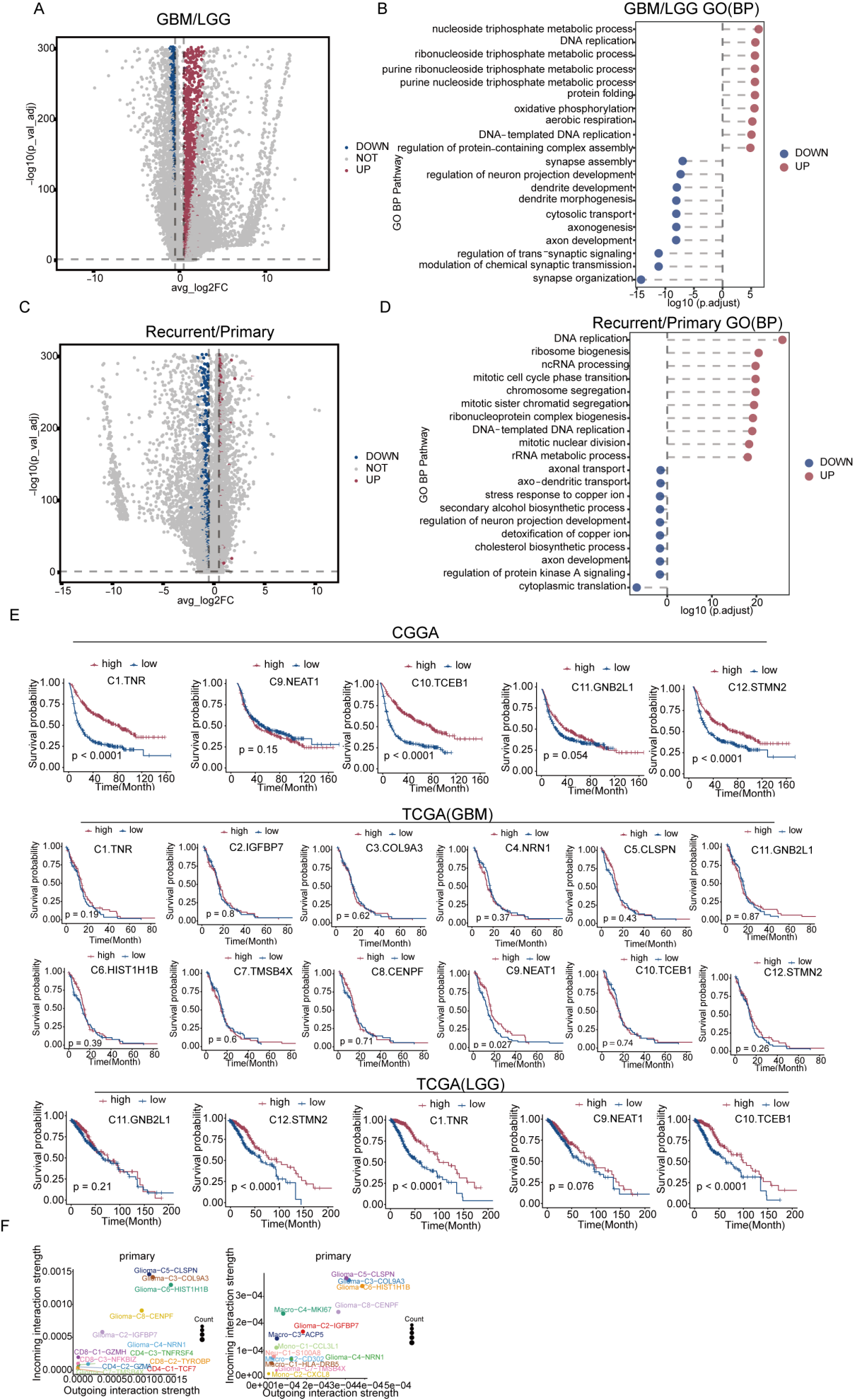
**

**Supplementary Figure S4: Identification of glioma cell clusters. (A)The volcano plot illustrating the Differentially Expressed Genes (DEGs) in glioma cells between glioblastoma multiforme(GBM) and low-grade glioma (LGG). (B)Gene Ontology (GO) enrichment analysis of DEGs between GBM and LGG. (C)The volcano plot illustrating the DEGs in glioma cells between primary and recurrent tumor. (D)GO enrichment analysis of DEGs between primary and recurrent tumor. (E)The Kaplan-Meier curve illustrating the relationship between the infiltration level of glioma cell clusters and patient survival prognosis in The Cancer Genome Atlas (TCGA) and Chinese Glioma Genome Atlas (CGGA) database. (F) Scatter plot analysis revealing communication patterns between glioma subpopulations and T cell/myeloid cell clusters in primary tumor.**

**
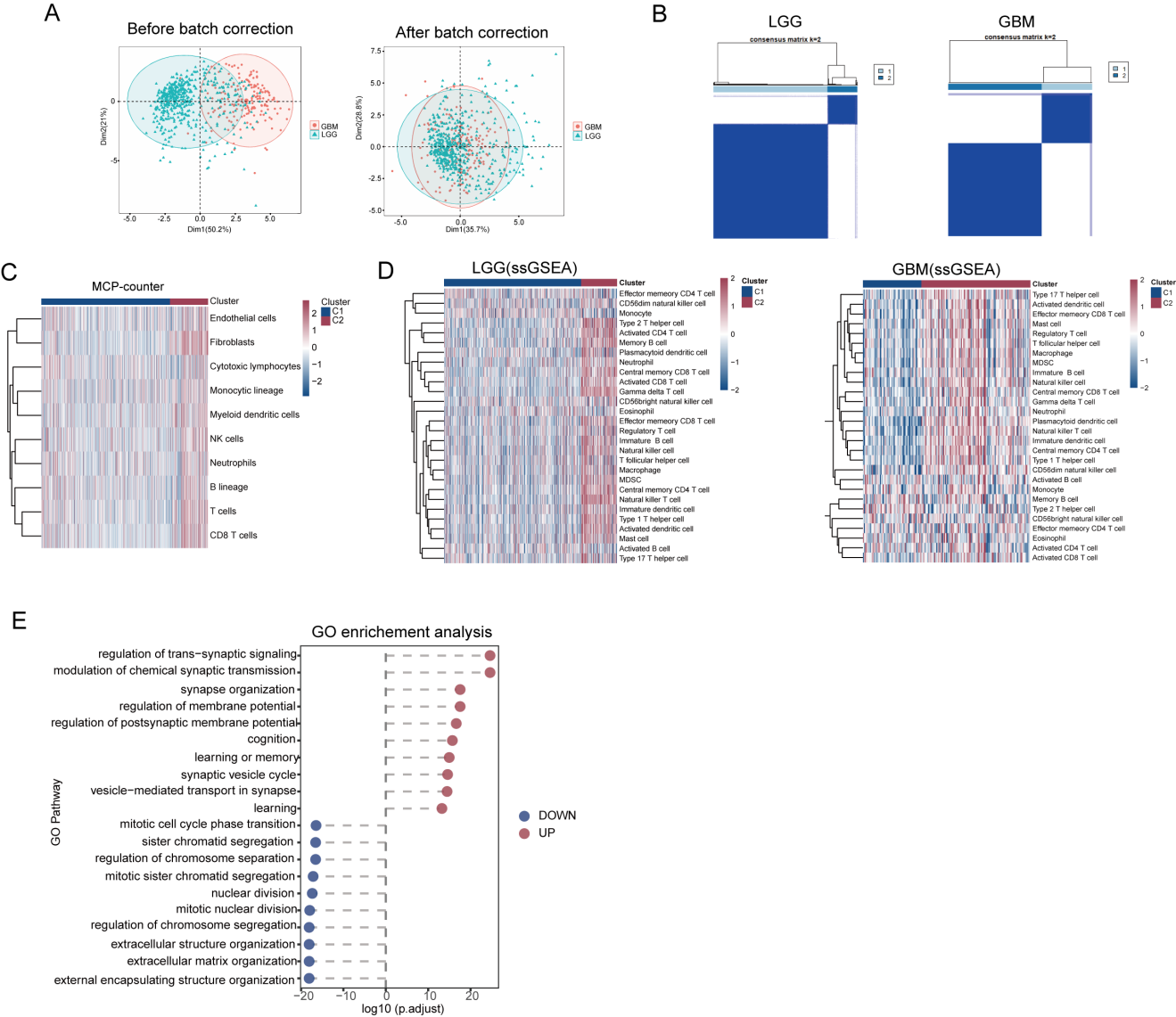
**

**Supplementary Figure S5: Identification of glioma subtypes. (A)Principal Component Analysis (PCA) visualization of batch effect removal of glioblastoma multiforme(GBM) and low-grade glioma (LGG) in The Cancer Genome Atlas (TCGA) database. (B)Heatmap showing consensus clustering of LGG and GBM in TCGA.(C)Heatmap showing immune cell score between C1 and C2 subtype in glioma. (D)Heatmap showing immune cell score between C1 and C2 subtype in LGG and GBM.(E)Gene Ontology (GO) enrichment analysis of Differentially Expressed Genes (DEGs) between C1 and C2 subtype.**

**
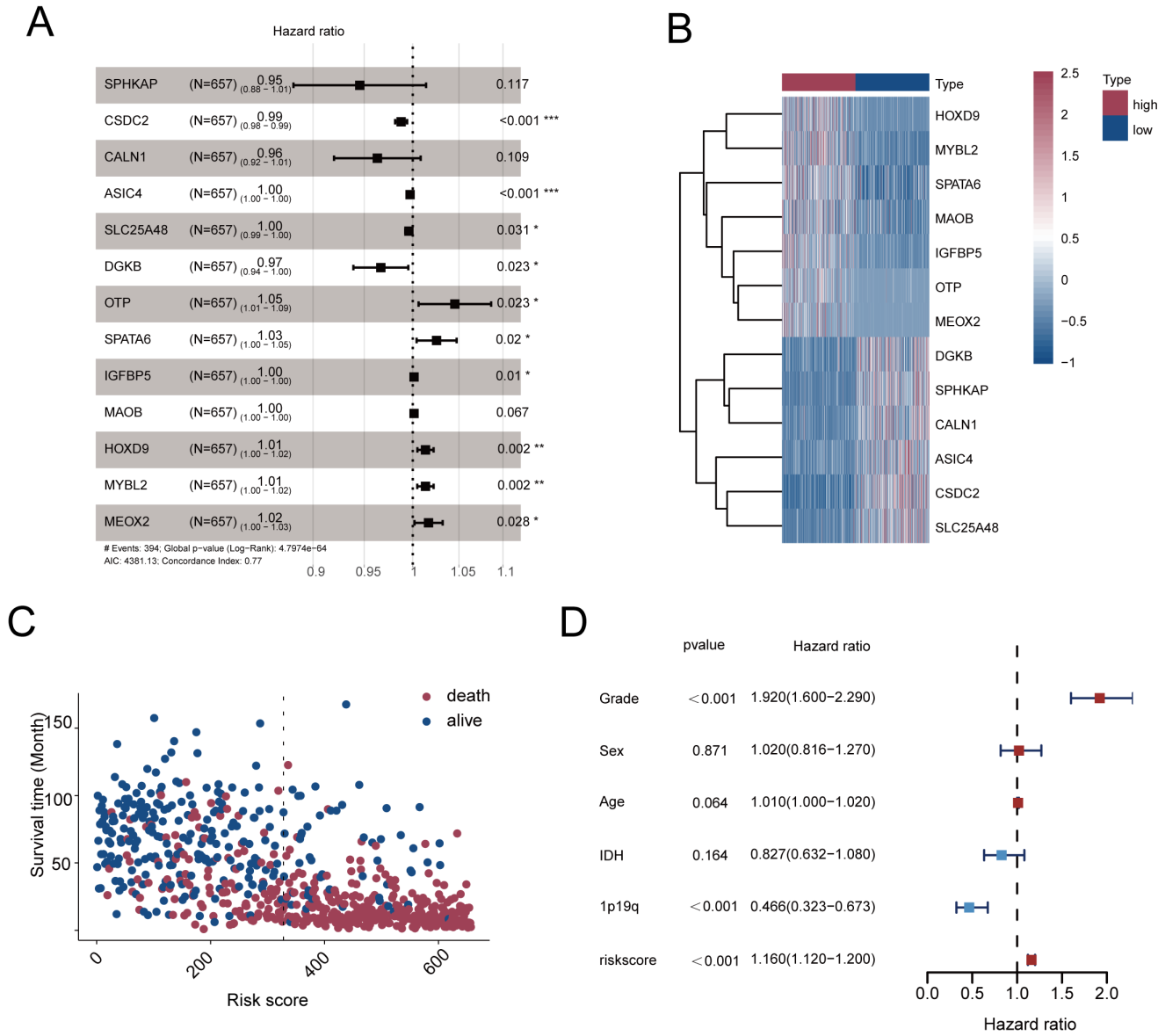
**

**Supplementary Figure S6: Construction of prediction model. (A) The forest plot displaying the hazard ratios (HRs) with 95% confidence intervals (CIs) for each gene included in the prognostic prediction model.*p<0.05, **p<0.01, ***p<0.001.(B) Heatmap showing the expression of genes in model between high and risk group. (C)Dotplot showing correlation of risk score and survival time and status in Chinese Glioma Genome Atlas (CGGA) datasets.(D)Multivariate Cox regression analysis of the risk score and clinical parameters.**
